# Supplementary material for: The Two-Photon Reversible Reaction of the Bistable Jumping Spider Rhodopsin-1
Source: Biophys J. 2019 Mar 5;116(7):1248–58. doi: 10.1016/j.bpj.2019.02.025 (PMC6451042; doi:10.1016/j.bpj.2019.02.025)
Supplement: Document S1. Supporting Materials and Methods, Figs. S1–S11, and Table S1 [file mmc1.pdf]

Biophysical Journal, Volume *116*

Supplemental Information

The Two-Photon Reversible Reaction of the Bistable Jumping Spider Rhodopsin-1

David Ehrenberg, Niranjan Varma, Xavier Deupi, Mitsumasa Koyanagi, Akihisa Terakita, Gebhard F.X. Schertler, Joachim Heberle, and Elena Lesca

Supplemental Material

Figure S1

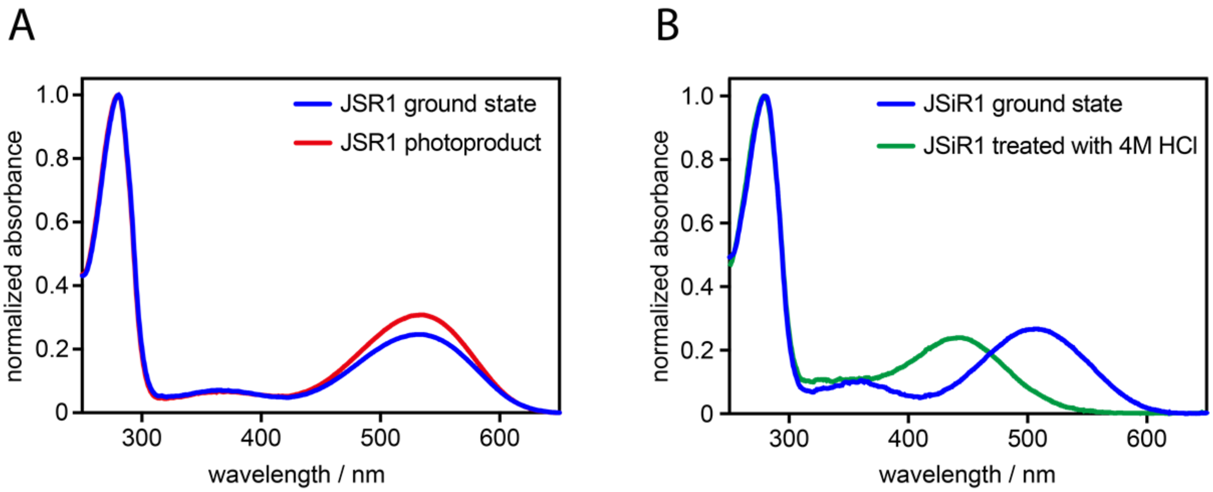

Normalized UV/Vis spectra of JSR1. **A)** JSR1 reconstituted with 11-*cis* retinal and of its photoproduct. They both have a maximum absorbance at 535 nm (16). **B)** UV/Vis absorption spectra of JSiR1 before and after acid denaturation. JSR1 reconstituted with 9-*cis* retinal was denaturated in the dark, resulting the typical ~440 nm maxima absorbance as indication of free Schiff base in solution (53).

Figure S2

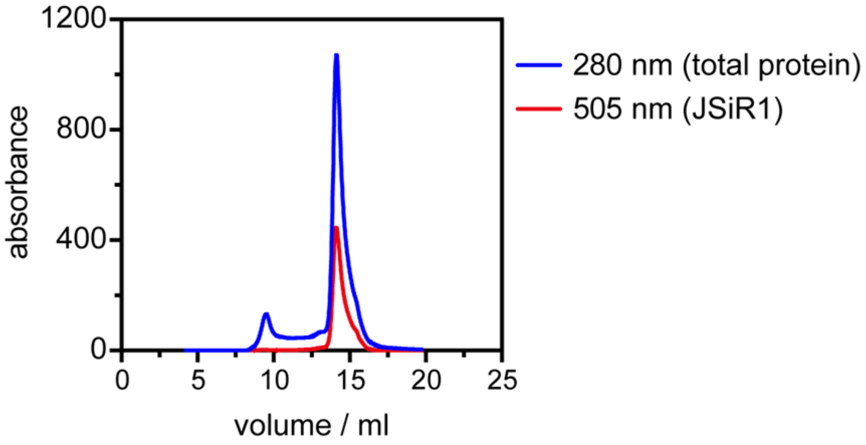

Purification of recombinant expressed JSiR1. Size-exclusion chromatography shows JSiR1 as a monodisperse sample. 280 nm absorbance (blue line) refers to the total protein amount, while 505 nm (red line) indicates the protonated Schiff base signal.

Figure S3

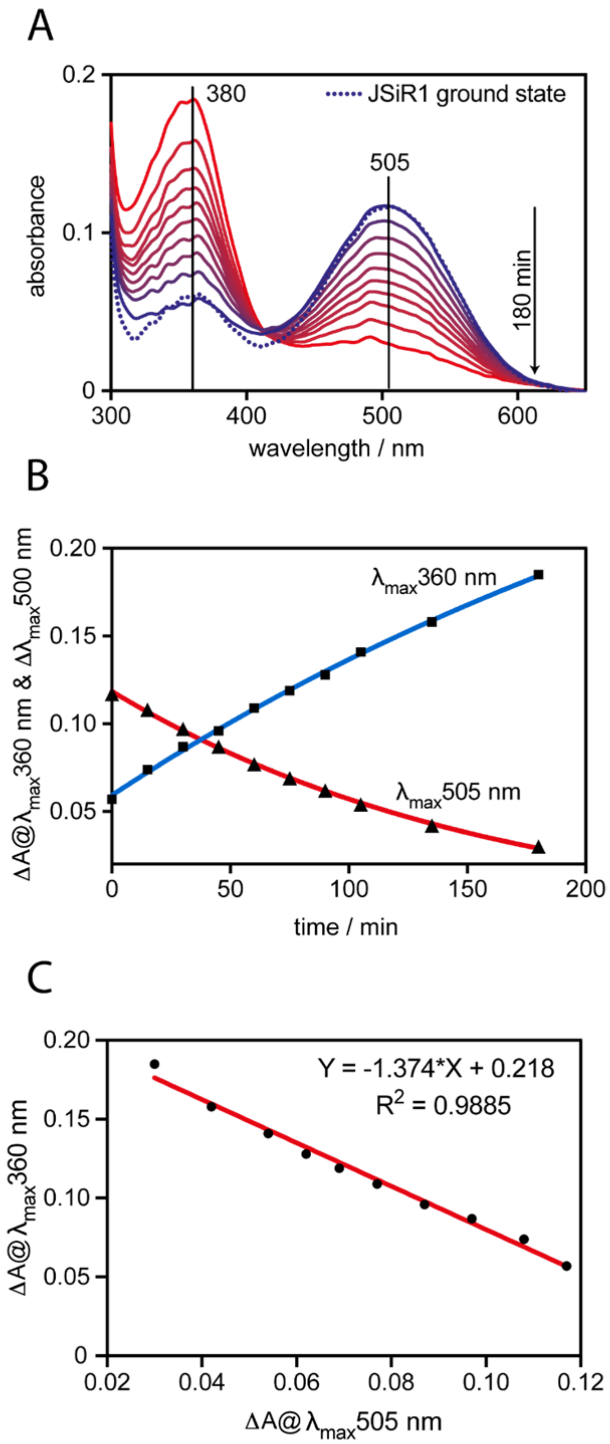

Extinction coefficient of JSiR1 in the opsin buffer. A) Hydroxylamine assay recorded over 3 hrs. The maxima absorbances at 360 nm (retinal) and 505 nm (JSiR1) are highlighted. **B)** The Δ360 and Δ505 are plotted against time (non-linear regression), showing a simultaneous increase/release of 9-*cis* retinal respectively **C)** Linear regression of Δ360 versus Δ505 shows the relation between increase/release of 9-*cis* retinal respectively. The slope corresponds to the Δ360/Δ505 = $\varepsilon_{retinal}/\varepsilon_{JSiR1}$, and has a value -1.37 ± 0.05.

Figure S4

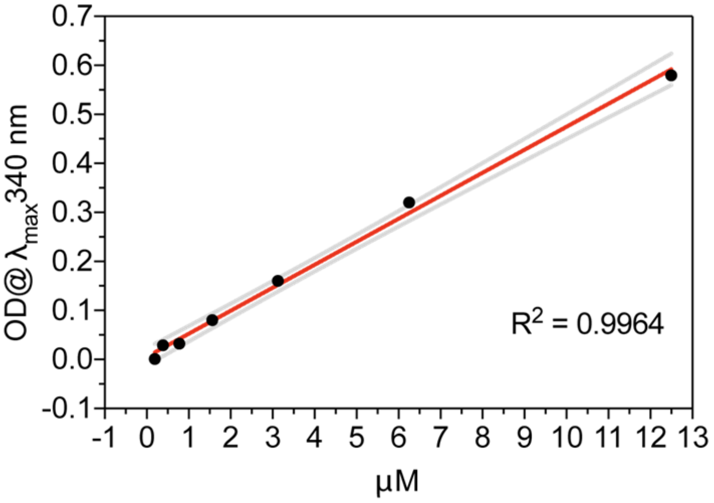

Standard curve of 9-*cis* retinal oxime in the opsin buffer. The $\varepsilon_{9\text{-}cis\ \text{retinal}}$ is 46930 M^{-1} cm^{-1}.

Figure S5

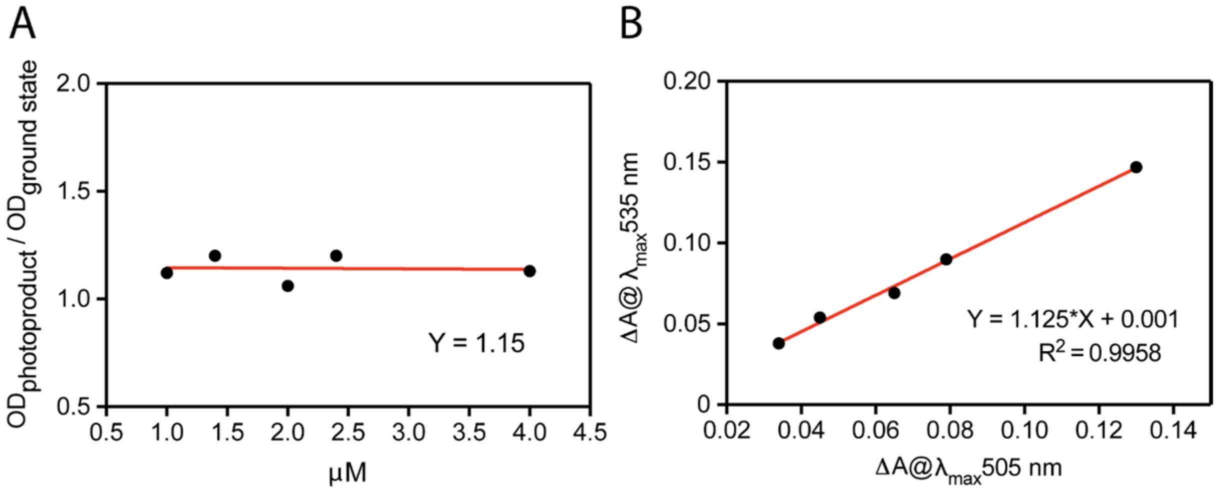

Extinction coefficient of JSR1 photoproduct in the opsin buffer. **A)** Interpolation of the ratio $OD_{photoproduct}/OD_{ground\ state}$ versus diverse concentration of JSiR1. The intercept corresponds to the $OD_{photoproduct}/OD = \varepsilon_{JSR1}/\varepsilon_{JSiR1}$= 1.15. **B)** Linear regression of OD max at 535 nm versus OD max at 505 nm. The slope corresponds to the $OD_{photoproduct}/OD_{ground\ state}$ and has a value 1.12 ± 0.04.

Figure S6

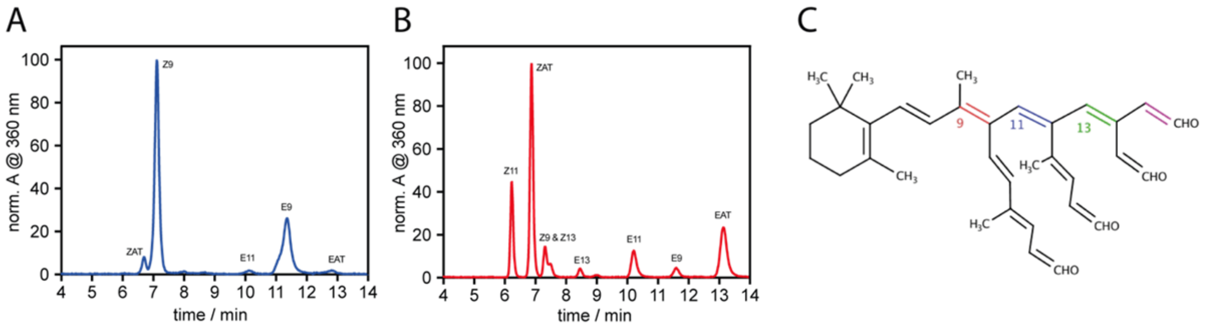

Chromophore configurations of JSiR1 before and after irradiation. HPLC chromatograms of retinal oximes (syn = Z, anti = E forms of retinal oximes) recorded at 360 nm and normalized **A)** JSiR1 ground state. **B)** JSR1 photoproduct. **C)** Chemical draw of retinal isomers.

The ground state of JSiR1 shows an interesting significative percentage of all-trans retinal (8%, Figure 2). Even if all the experiments were under controlled 640 nm light, we cannot exclude light-independent isomerization phenomena (e.g. thermal isomerisation) and, in fact, similar cases have been already reported. For instance, mouse melanopsin —30% similar to JSR1— shows thermal isomerization at 37˚C(1). Another example is the mekada TMT1A opsin, that also displays an heterogenous composition in the ground state (all *trans*, 9-*cis* and 13-*cis* retinal) (2).

1. Tsukamoto, H., Y. Kubo, D.L. Farrens, M. Koyanagi, A. Terakita, and Y. Furutani. 2015. Retinal attachment instability is diversified among mammalian melanopsins. J. Biol. Chem. 290: 27176–27187.
2. Sakai, K., T. Yamashita, Y. Imamoto, and Y. Shichida. 2015. Diversity of active states in TMT opsins. PLoS One. 10: 1–12.

Figure S7

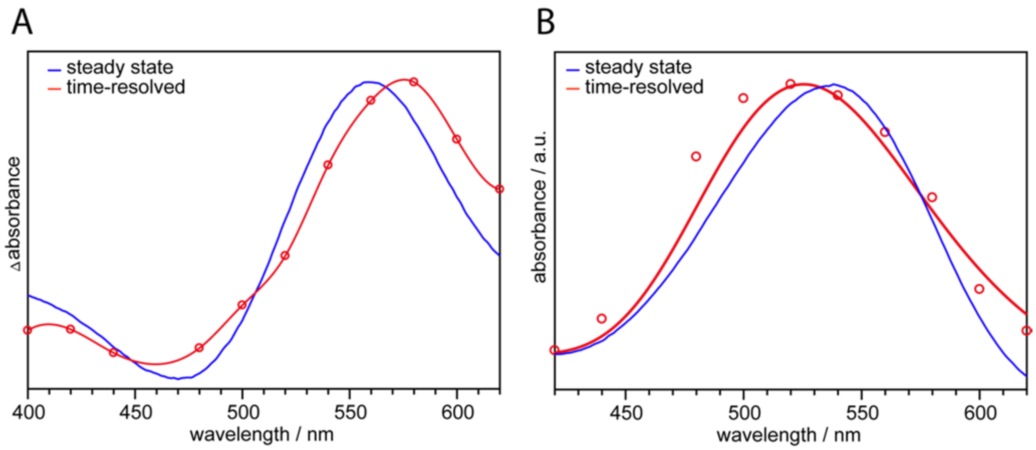

Comparison of Flash Photolysis with Steady State data. A) The shape of the final spectrum of the time-resolved dataset (red) resembles that of Meta-minus-Iso (blue). **B)** The absolute spectrum of a-Meta obtained by addition of the steady state spectrum of Iso to the time-resolved dataset has a slightly different shape compared to the Meta/Rho spectrum after illumination. This could indicate a different *syn*/*anti* composition due to the laser pulses. The difference in absorption maxima is less than 10 nm.

Figure S8

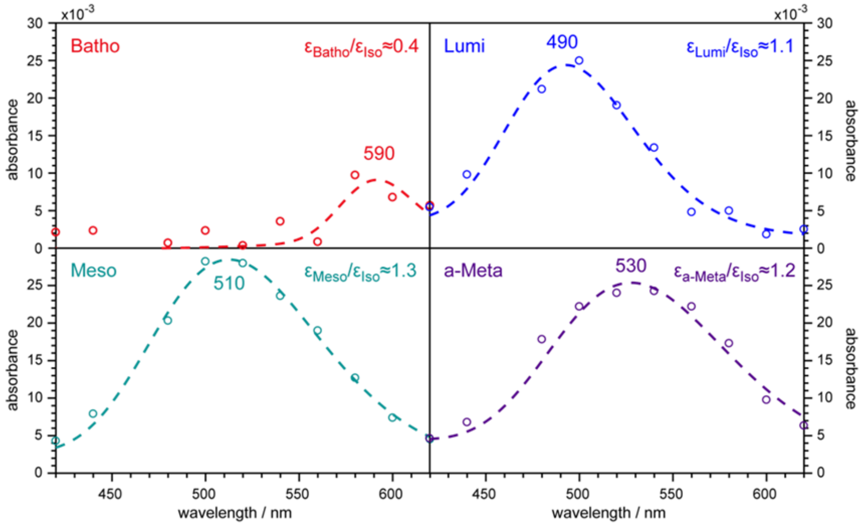

Calculated absolute spectra of the photoproducts in the forward reaction.

Absolute spectra of the intermediates in the forward photoreaction Iso to Meta. These were obtained by eliminating the negative contribution of Iso in the difference dataset.

It has to be noted that maxima in difference signals might not represent the real absorption maximum of the intermediate species due to spectral overlap of the initial state and the photoproduct. To determine the real absorption maxima of the respective intermediate states, the negative contribution of the initial Iso state in the dataset of the forward reaction must be subtracted. This is achieved by adding the UV/Vis spectrum of Iso in such a way that yields only positive absorption (58). Since the Meta state concludes the time-resolved dataset, the addition of the Iso spectrum was done by reproducing the spectral shape of the steady-state Meta spectrum obtained via UV/Vis spectroscopy (Figure S7B, a non-linear least squares regression was performed in MATLAB to approximate the steady-state spectrum of the illuminated sample (Figure S3B)). The resulting spectra of Batho, Lumi and Meso show absorption maxima at 590, 490 and 510 nm, respectively (Figure 4). Comparing the maximum absorption of the calculated intermediate spectra with the added Iso spectrum yields extinction coefficient ratios, which are typical for bistable pigments (59) except for the low extinction coefficient for Batho. The extinction coefficient ratio $\varepsilon_{Meta}/\varepsilon_{Iso}$ of 1.2 is in accordance the previously found value of 1.15 (see "Features of JSR isorhodopsin (JSiR1)") confirming our approach.

Figure S9

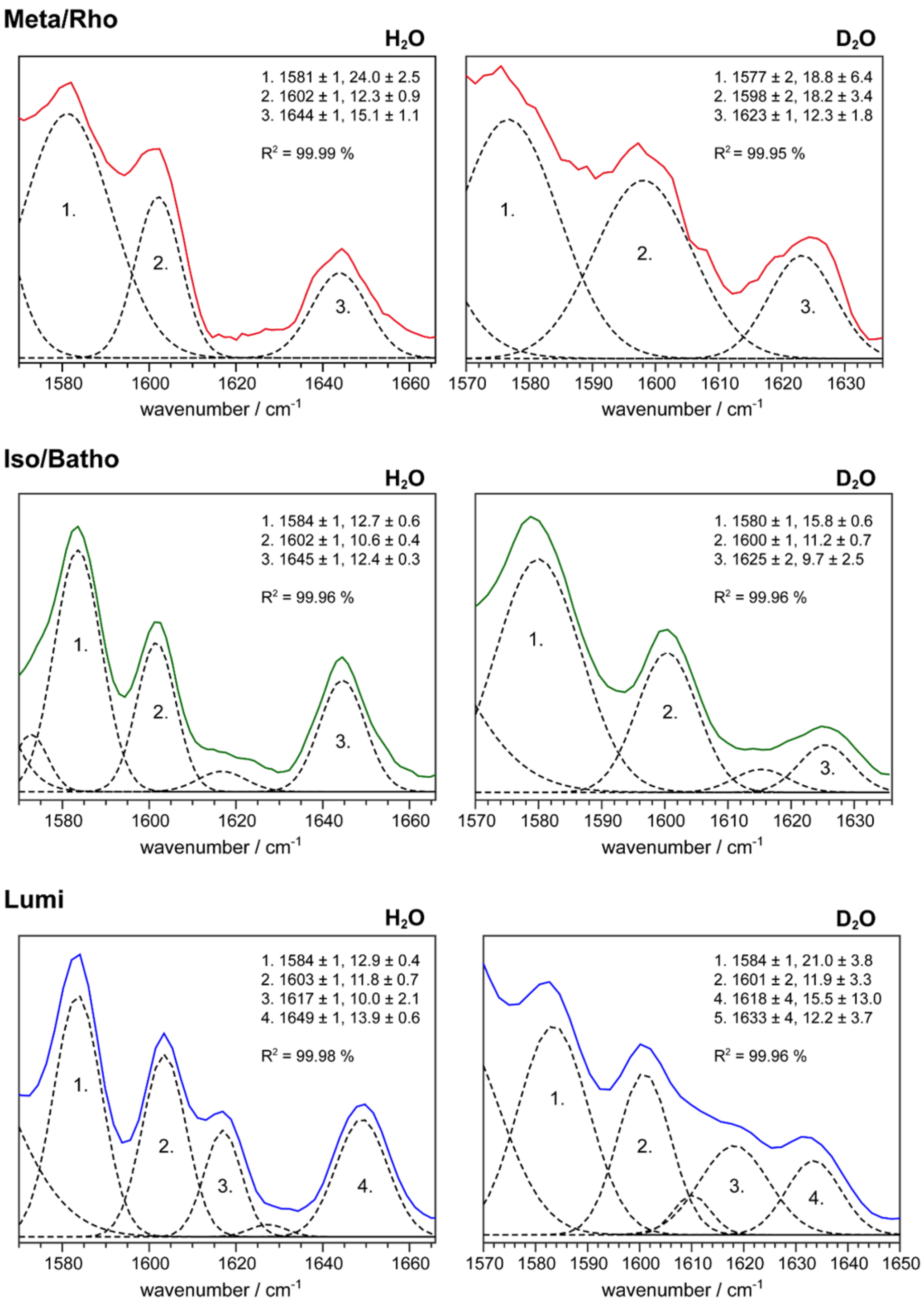

Fits of the Schiff Base region.

The dashed lines represent the fits with Gaussian functions on the raw data. R-squared was for all fits above 99.95 %. Fit parameters with corresponding error are given in the top right corner.

Figure S10

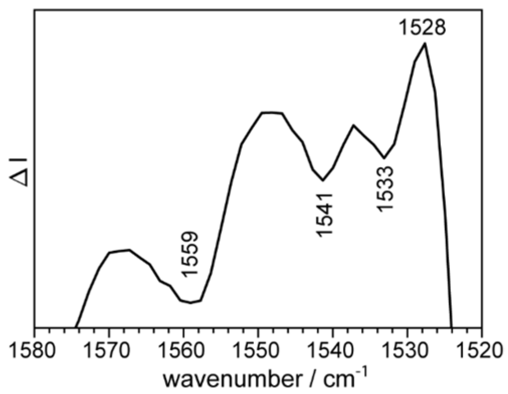

Resonance Raman difference spectra of Meta/Rho minus Isorhodopsin in the C=C stretch region
Spectra of Figure 7 were normalized to band at 1008 cm^{-1} prior the subtraction. The resulting band pattern resembles the FTIR difference spectra of Figure 8.

Figure S11

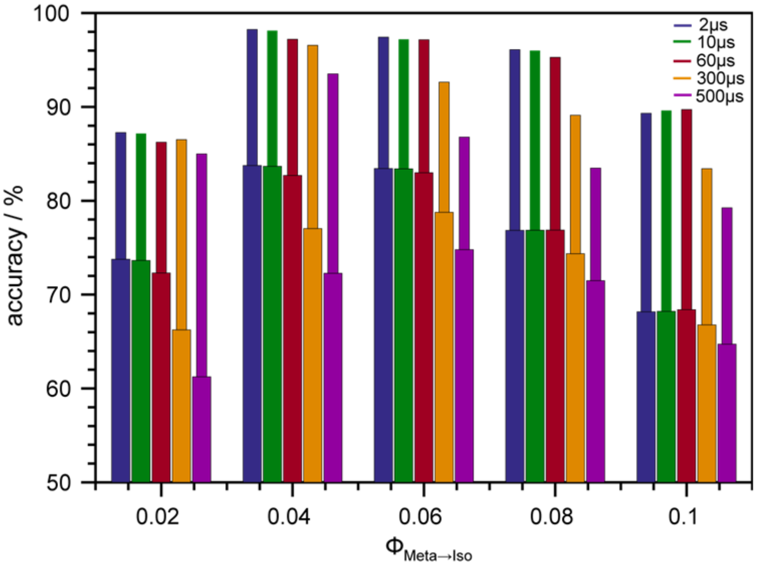

Photostationary state simulation.

The photolysis rate depends on the quantum yield(s) of a certain state in the photocycle of JSR1 as described in “Methods”. This value is unknown for Rho and Meta, as well as for other possible transitions from Meta-to-Iso. Hence, these values were varied from 0.5 to 0.9 and from 0.2 to 0.7, respectively. For each combination of these quantum yields the system of ODEs were solved for different time constants of the photoreverse reaction, i.e. from Meta to Rho. The plot shows the best scenario (thinner bar) and the overall performance (all combinations of quantum yields; wider bar) for a given Meta to Iso quantum efficiency and time constant for the photoreverse reaction. Best results are achieved for quantum efficiencies between 0.04 and 0.06 as well as for time constants below 60 µs. For larger time constants, the accuracy starts to drop significantly.

The table S1 grants a more detailed view of other parameters, which are grouped into the wider bar. An accuracy above 90% is considered to be in accordance with the HPLC data due to the experimental error in the determination of the retinal isomers and are highlighted in green for $\Phi_{Rho \rightarrow Iso}$ = 0.04 and in blue for 0.06. The corresponding value is the fraction of Φ_{Rho}/ϕ_{Meta}. For $\Phi_{Rho \rightarrow Iso}$ = 0.04 a fraction of at least 2 is needed in order to reproduce the HPLC results while for 0.06 the average fraction is 1.6. This value is in accordance to literature values for other bistable opsins. Overall, it can be stated that it is a prerequisite for Meta accumulation that the quantum yield of Meta is lower than that of Rho and that the photoreverse reaction (Meta -> Rho) has to be faster than the photoactivation (Rho -> Meta).

Table S1

Φ_{Rho}/ϕ_{Meta}	**0.2**	**0.3**	**0.4**	**0.5**	**0.6**	**0.7**
**0.5**	2.5	1.7	1.3	1	0.8	0.7
**0.6**	3	2	1.5	1.2	1	0.9
**0.7**	3.5	2.3	1.7	1.4	1.2	1
**0.8**	4	2.7	2	1.6	1.3	1.1
**0.9**	4.5	3	2.3	1.8	1.5	1.3

Green: 0.04, blue: 0.06
