# Supplementary material for: The Two-Photon Reversible Reaction of the Bistable Jumping Spider Rhodopsin-1
Source: Biophys J. 2019 Mar 5;116(7):1248–58. doi: 10.1016/j.bpj.2019.02.025 (PMC6451042; doi:10.1016/j.bpj.2019.02.025)
Supplement: Document S2. Article plus Supporting Material [file mmc2.pdf]

Biophysical Journal
Article

Biophysical Society

# The Two-Photon Reversible Reaction of the Bistable Jumping Spider Rhodopsin-1

David Ehrenberg,[1] Niranjan Varma,[2,5] Xavier Deupi,[3] Mitsumasa Koyanagi,[4] Akihisa Terakita,[4] Gebhard F. X. Schertler,[2,5,*] Joachim Heberle,[1,*] and Elena Lesca[2,5,*]

[1]Experimental Molecular Biophysics, Department of Physics, Freie Universität Berlin, Berlin, Germany; [2]Division of Biology and Chemistry-Laboratory for Biomolecular Research and [3]Division of Neutrons and Muons-Laboratory for Scientific Computing and Modelling, Paul Scherrer Institute, Villigen, Switzerland; [4]Department of Biology and Geosciences, Graduate School of Science, Osaka City University, Osaka, Japan; and [5]Department of Biology, ETH Zürich, Zürich, Switzerland

ABSTRACT Bistable opsins are photopigments expressed in both invertebrates and vertebrates. These light-sensitive G-protein-coupled receptors undergo a reversible reaction upon illumination. A first photon initiates the *cis* to *trans* isomerization of the retinal chromophore—attached to the protein through a protonated Schiff base—and a series of transition states that eventually results in the formation of the thermally stable and active Meta state. Excitation by a second photon reverts this process to recover the original ground state. On the other hand, monostable opsins (e.g., bovine rhodopsin) lose their chromophore during the decay of the Meta II state (i.e., they bleach). Spectroscopic studies on the molecular details of the two-photon cycle in bistable opsins are limited. Here, we describe the successful expression and purification of recombinant rhodopsin-1 from the jumping spider *Hasarius adansoni* (JSR1). In its natural configuration, spectroscopic characterization of JSR1 is hampered by the similar absorption spectra in the visible spectrum of the inactive and active states. We solved this issue by separating their absorption spectra by replacing the endogenous 11-*cis* retinal chromophore with the blue-shifted 9-*cis* JSiR1. With this system, we used time-resolved ultraviolet-visible spectroscopy after pulsed laser excitation to obtain kinetic details of the rise and decay of the photocycle intermediates. We also used resonance Raman spectroscopy to elucidate structural changes of the retinal chromophore upon illumination. Our data clearly indicate that the protonated Schiff base is stable throughout the entire photoreaction. We additionally show that the accompanying conformational changes in the protein are different from those of monostable rhodopsin, as recorded by light-induced FTIR difference spectroscopy. Thus, we envisage JSR1 as becoming a model system for future studies on the reaction mechanisms of bistable opsins, e.g., by time-resolved x-ray crystallography.

## INTRODUCTION

Animal opsins are light-sensitive G-protein-coupled receptors (GPCRs) mainly involved in vision and circadian clock entrainment (1). In these GPCRs, photon absorption results in the isomerization of a retinal chromophore covalently linked to the protein through a protonated Schiff base. Retinal isomerization leads to structural changes in the receptor, resulting in the recruitment and activation of G-proteins and other downstream signaling cascades.

According to the stability of the photoactivated state, opsins can be classified as monostable or bistable. In monostable opsins, deprotonation and subsequent hydrolysis of the Schiff base during Meta II decay leads to the eventual loss of the retinal chromophore (bleaching). Once released, the all-*trans* retinal is reisomerized in the retinal pigment epithelium and reconstituted into an opsin obtaining a functional rhodopsin (2). In contrast, in bistable opsins—such as jumping spider rhodopsin-1 (JSR1) (3)—retinal remains in the protein binding pocket throughout the entire photoreaction (4), and its isomerization leads to the formation of a thermally stable active state (acid-Meta) (4–7), suggesting the presence of a protonated Schiff base (4,6). Illumination of this state recovers the original inactive ground state (Rho). Hence, these bistable opsins exhibit both forward and backward photoreactions, i.e., the retinal isomerizes back and forth between *cis* and *trans* configurations upon repeated illumination (7).

The fast rate of these photoreactions hinders our ability to understand the activation mechanism of bistable opsins at the molecular level. Previous studies on squid and octopus rhodopsins revealed numerous intermediates in the forward

*Submitted September 21, 2018, and accepted for publication February 19, 2019.*

*Correspondence: gebhard.schertler@psi.ch or joachim.heberle@fu-berlin.de or elena.lesca@psi.ch

David Ehrenberg and Niranjan Varma contributed equally to this work.

Editor: Andreas Engel.

https://doi.org/10.1016/j.bpj.2019.02.025

© 2019 Biophysical Society.

This is an open access article under the CC BY license (http://creativecommons.org/licenses/by/4.0/).

reaction of the cycle (all-*trans* half-cycle: Rho > Batho > Lumi > Meso > acid-Meta), and an additional state between Meso and Meta (t-Meta) has been proposed for octopus rhodopsin (8–10). However, insights about the backward reaction—11-*cis* half-cycle—are scarce. In octopus rhodopsin, this reaction has been suggested to consist of only two detectable intermediates (acid-Meta > $I_1$ > $I_2$ > Rho) and to be considerably slower than the forward reaction (11). In the case of squid rhodopsin, it has been suggested that the Schiff base de- and reprotonates during the recovery of the ground state (12).

There are several issues that complicate spectroscopic studies of bistable opsins, such as being able to disentangle the similar absorption spectra of the ground and active states or the absence of a model system—like bovine rhodopsin for monostable opsins (2)—that can be recombinantly expressed and stably purified to yield large quantities of functional protein (13). To date, bistable opsins for biophysical studies are sourced from native retinae, precluding protein engineering and detailed investigations (14–17).

Here, we report the biochemical and biophysical characterization of recombinantly produced JSR1, a potential model for bistable opsins. JSR1 is a green-light sensitive visual pigment found in the rhabdomeric photoreceptor cells of *Hasarius adansoni* (3). The endogenous chromophore of JSR1 is the 11-*cis* retinal isomer, with an absorbance maximum at 535 nm in the ground as well as in the all-*trans* photoproduct Meta state (3). To improve the spectroscopic separation between inactive and active states of the protein, we used the 9-*cis* retinal analog because of its hypsochromic shift (isorhodopsin, JSiR1). Furthermore, we have characterized the forward and backward photoreactions of JSiR1 under ambient conditions using time-resolved ultraviolet-visible (UV-Vis) spectroscopy, identifying different intermediates and their time constants of rise and decay. Additionally, vibrational spectroscopy provided evidence for structural changes of JSiR1 upon photoactivation on the level of single bonds.

This detailed characterization of the photoactivation process of JSR1 sheds light on the mechanistic basis of bistability in light-sensitive GPCRs. We also expect that these results will assist in establishing JSR1 as a model system for the study of novel light-controlled molecular switches in optogenetic applications.

## METHODS

### Cloning and expression

The cDNA of JSR1 (18) from *Hasarious adansoni* (AB251846) was cloned into a pcDNA 3.1(+) vector (Thermo Fisher Scientific, Carlsbad, CA) and stably expressed in HEK 293 GnTI− (gift from Khorana (19)) in Dulbecco's modified Eagle's medium (Thermo Fisher Scientific) supplemented with 10% fetal bovine serum and G418 (Thermo Fisher Scientific) as a selection marker. The stable clones were adapted to suspension systems and cultured in FreeStyle 293 Expression Medium (Thermo Fisher Scientific), reaching a final cell count of 2.5–3 × $10^6$ cell/mL. The cultures were then harvested by centrifugation at 10,600 × *g* for 10 min at 4°C.

### Membrane preparation and protein purification

The centrifuged cells were resuspended in 50 mM HEPES, 140 mM NaCl, and 3 mM $MgCl_2$ with protease inhibitors (Roche cOmplete EDTA-free Protease Inhibitor Cocktail; Sigma-Aldrich, Buchs, Switzerland) and mechanically homogenized. The membrane suspension was reconstituted with 9-*cis* retinal (98% grade; Sigma-Aldrich) or 11-*cis* retinal (98% grade, from National Eye Institute, National Institutes of Health, Bethesda, MD) for 16 h at 4°C, after which it was solubilized with n-dodecyl-$\beta$-D-maltopyranoside (DDM Anagrade; Anatrace, Maumee, OH) for 2 h at 4°C. The solubilized membranes were centrifuged at 100,000 × *g* for 1 h at 4°C. The supernatant was then supplemented with cyanogen bromide-activated Sepharose 4B resin (GE Healthcare Life Science, Freiburg, Germany) bound with anti-1D4 antibody (Cell Essentials, Boston, MA). The resultant slurry was loaded onto a BioRad Glass Econo-Column (BioRad, Hercules, CA) and washed off with 50 mM HEPES, 140 mM NaCl, and 0.195 mM DDM (opsin buffer). Protein elution was carried out overnight at 4°C in opsin buffer with 800 $\mu$M 1D4 elution peptide (Peptide 2.0, Chantilly, VA). The eluted receptor was concentrated using an Amicon Ultra 100 kDa (MilliporeSigma, Burlington, MA) concentrator in a bench top centrifuge at 1083 × *g* at 4°C. The concentrated protein was loaded onto a Superdex 200 Increase 10/300 GL (GE Healthcare Life Science) SEC column using an FPLC system (Äkta Explorer 10; GE Healthcare Life Science). The main fractions containing the receptor were pooled and concentrated. The sample preparation was further assessed by sodium dodecyl sulphate-polyacrylamide gel electrophoresis (SDS-PAGE) and UV-Vis spectroscopy. The purification and the other experiments have been performed under 640 nm light.

### UV-Vis spectroscopy, illumination, and acid denaturation assay

A commercially available spectrometer (UV-2401PC; Shimadzu, Kyoto, Japan) was used to record spectra of JSiR1 samples at 20°C. For all the experiments reported here, purified JSiR1 was used. Purity is assed with the optical density (OD) 280/505 nm ($\lambda_{max}$) ratio, where 2.5–2.8 was observed. SDS-PAGE gels and size-exclusion chromatogram traces were used to evaluate sample quality. Protein concentration was calculated by the Beer-Lambert law, where $\varepsilon_{JSiR1}$ is ~32.660 $M^{-1}$ $cm^{-1}$ (see below), and the molecular weight was calculated from the protein sequence as 44 kDa. Illumination was carried out in the dark with 495 nm (low-pass filter) on a projector lamp at maximal intensity for 10 min (lamp parameters 150 W, 2 A, 50/60 Hz, 220–240 V, irradiance 5 W/$cm^2$). In the acid denaturation assay, purified JSiR1 at ~OD 0.05 was supplemented with 4.275 M HCl, and a spectrum was recorded soon after (20).

### Extinction coefficient of 9-*cis* retinal oxime

The extinction coefficient of the 9-*cis* retinal oxime was determined in the opsin buffer as described. A standard curve was calculated with different concentration of 9-*cis* retinal oxime. 1 M hydroxylamine was dissolved in the opsin buffer (opsin buffer H). This solution was also used to baseline the spectrophotometer. A stock of 4 mM 9-*cis* retinal was created in the opsin buffer and incubated for 20 min in ice to complete the oxime formation. From this stock, 10 dilutions of 9-*cis* retinal oxime were created in opsin buffer H (100, 50, 25, 12.5, 6.25, 3.12, 1.56, 0.78, 0.39, 0.19 $\mu$M), and their absorbance was measured. Collected data were interpolated where the slope of the line equals the molar absorptivity coefficient ($\varepsilon \times$ l, where l is the pathlength), and therefore $\varepsilon$ = slope/l (Fig. S4).

## Extinction coefficient of JSiR1 and hydroxylamine assay

JSiR1 in opsin buffer was treated with 300 mM hydroxylamine at final 0.119 OD. The sample with hydroxylamine was measured at different time points for 3 h at 20°C.

$\Delta 360$ is defined as the difference in absorbance of "hydroxylamine-treated sample—ground state" (how much retinal is released), and $\Delta 505$ is the difference in absorbance of "ground state—hydroxylamine-treated sample" (amount of opsin bleached). Assuming that one molecule of JSiR1 releases one molecule of 9-*cis* retinal, $\Delta 360/\Delta 505 = \varepsilon_{retinal}/\varepsilon_{JSiR1}$, and therefore $\varepsilon_{JSiR1} = (\Delta 505 \times \varepsilon_{retinal})/\Delta 360$, where $\varepsilon_{retinal}$ is 46,930 $M^{-1}$ $cm^{-1}$, as calculated above (Fig. S3). $\Delta 360$ and $\Delta 505$ are plotted against time, showing a simultaneous increase (or release) of 9-*cis* retinal, respectively. One-phase association nonlinear regression was used for $\Delta 360$, whereas one-phase decay was used for $\Delta 505$ (Fig. S3 *B*). The linear regression between $\Delta 360/\Delta 505$ calculates the slope of the intercept (Fig. S3 *C*). This method is based on (21,22).

## Extinction coefficient of JSR1 photoproduct

Upon illumination, JSiR converts into a stable and heterogeneous photoproduct (JSR1 photoproduct), as shown by HPLC analysis (see Results). Therefore, $OD_{ground\ state}/\varepsilon_{JSiR1} = OD_{photoproduct}/\varepsilon_{photoproduct}$, and so $\varepsilon_{photoproduct} = (OD_{photoproduct} \times \varepsilon_{ground\ state})/OD_{ground\ state}$. To validate this reasoning, the ratio $OD_{photoproduct}/OD_{ground\ state} = \varepsilon_{photoproduct}/\varepsilon_{ground\ state}$ has been calculated and plotted through different sample concentrations (4.0, 2.4, 2.0, 1.4, and 1.0 $\mu$M) (Fig. S5). In addition, the slope of intercept corresponds to $OD_{photoproduct}/OD_{ground\ state}$ (Fig. S5).

## HPLC

Extraction of the chromophore from non- and irradiated JSiR1 was based on Groenendijk et al. (23) and analyzed by high-performance liquid chromatography (HPLC, Hewlett Packard Series 1050, diode array detector; Hewlett Packard, Palo Alto, CA). About 100 $\mu$L of purified JSiR1 at OD 0.2 was treated with 250 $\mu$L 20% cold methanol and then with 50 $\mu$L 1 M hydroxylamine ($NH_2OH$) (pH 7.0) and incubated on ice for 10 min. Finally, n-hexane (400 $\mu$L) was added to separate retinal oximes from the rest. The sample was aliquoted in dark glass vials, injected onto a YMC-Pack silica column (particle size 3 $\mu$m, volume 150 × 6.0 mm), and eluted with n-hexane containing 15% ethyl acetate and 0.15% ethanol at a flow rate of 1.0 mL/min. Data were recorded by a diode array detector at 360 nm. The results were validated through comparison with literature and internal calibration curves of diverse retinal oximes. The area under the peaks was calculated with baseline correction in Origin (OriginLab, Northampton, MA). Area under the peaks values were multiplied by the relative oxime extinction coefficient (24) to obtain a correct value. All the plots were created with GraphPad Prism, and MarvinView was used to draw the retinal isomers.

## Time-resolved UV-Vis spectroscopy

Time-resolved UV-Vis experiments on JSR1 reconstituted with 9-*cis* retinal (JSiR1, isorhodopsin) solubilized in 0.195 mM DDM, 50 mM HEPES, 140 mM NaCl (pH 6.5) were performed using a commercial flash photolysis setup (LKS70; Applied Photophysics, Leatherhead, UK) essentially as described in (25). Briefly, the photoreaction was induced by a short laser pulse emitted by a neodymium yttrium aluminum garnet laser (Quanta-Ray; Spectra-Physics, Santa Clara, CA), which drives an optical parametric oscillator. The emission (10 ns pulse width) was set to a wavelength of 465 nm and an energy density 3–4 mJ/$cm^2$ at the sample, leading to a photoconversion of ~6–9% of JSiR1 (as determined by UV-Vis spectroscopy; data not shown). Single-shot absorption changes were recorded in a range from 400 to 620 nm in 20 nm steps (omitting 460 nm due to scattering by the photolyzing laser) on two timescales, with the faster time range (<1 ms) recorded with the light source (Xe arc lamp) in pulsed mode and the slower timescale (>10 $\mu$s) recorded with the lamp running in continuous operation. The data were merged to cover the timescale from 50 ns to 1 s. Because the exciting laser flash irreversibly photoconverts a fraction of the initial proteins with 9-*cis* retinal to the all-*trans* Meta state, data were recorded in both wavelength directions, i.e., from 400 to 620 nm and vice versa. A fresh sample was used for each direction to obtain meaningful data of the Iso-to-Meta transition. After 11 laser flashes (requirement for one wavelength scan), the accumulation of Meta raised to ~60%, which was proportionally subtracted from the measured data. The resulting two data sets were intensity corrected and merged with weighting factors for each wavelength, taking account of the varying contribution of the Iso-to-Meta transition in the data set. All of this was achieved by using a nonlinear least squares regression, expecting the data set to finish with a difference spectrum of Meta-minus-Iso (Fig. S3 *A*).

For experiments on the Meta state, the sample was illuminated by a 473 nm diode-pumped solid-state laser (30 mW; CNI, Changchun, China) for 5 min before the experiment. All measurements were performed at 20°C using a circulating water bath (F25; JULABO, Seelbach, Germany). For the global fit of the illuminated sample, we used a model of two parallel photoreactions, one representing the forward reaction with fixed time constants (Fig. 4 *B*, *bottom panel*, *gray lines*) and the other with indefinite time constants for the backward reaction. All calculations were done in MATLAB (The MathWorks, Natick, MA).

## FTIR spectroscopy

A highly concentrated JSiR1 at pH 6.5 sample was dried on a $BaF_2$ window and sealed by a second window. The protein film was rehydrated via the saturated vapor phase of a glycerol/water mixture (5:5 wt/wt). Light-induced infrared (IR) difference spectra were recorded in transmission mode using a commercial Fourier Transform infrared (FTIR) spectrometer (Vertex 80v; Bruker, Billerica, MA) as described in (25), running at a spectral resolution of 2 $cm^{-1}$. A spectrum was taken while keeping the sample in the dark as a background. Subsequently, spectra were recorded after 5 min of illumination by a light-emitting diode (LED) emitting at 470 nm.

## Resonance Raman spectroscopy

Resonance Raman spectroscopy was performed on a LabRAM spectrometer (JobinYvon/HORIBA, Kyoto, Japan) as described in (26) but using frequency-stabilized diode-pumped solid-state lasers (CNI) that emit at 457 or 532 nm, respectively. Before the Raman recordings with the rotational cuvette, 200 $\mu$L of concentrated JSiR1 at pH 6.5 was illuminated by a 470 nm LED. For deuterium exchange, the sample was rigorously washed with $D_2O$ containing 50 mM HEPES, 140 mM NaCl, and 0.195 mM DDM. Low-temperature experiments were done in a cryostage (TMHS500; Linkam, Tadworth, UK). Concentrated JSiR1 was dried on a silicon crucible, rehydrated with 4 $\mu$L of an $H_2O$/glycerol or $D_2O$/glycerol mixture via the vapor phase and sealed with a glass coverslip using vacuum paste. The sample was carefully protected from light until the desired temperature was reached.

## Photostationary state simulation

The population of a photocycle intermediate depends on the photoreaction upon absorption of a photon by the parent state (photolysis rate $k_{ph,x}$), its thermal decay rate $k_{th,x}$, and on the concentration of its precursor. As examples for Rho,

$$\frac{d[Rho]}{dt} = -k_{ph,Rho} \times [Rho] \times t + k_{th,M2} \times [M2] \times t,$$

$$k_{ph,x} = \ln(10) \times \frac{\Phi_x \times \varepsilon_x}{N_A \times h \times v} \times I,$$

where the photolysis rate $k_{ph,x}$ depends on the quantum efficiency $\Phi_x$, the extinction coefficient $\varepsilon_x$, the frequency $v$, and the irradiance of the incoming light in W/cm$^2$ (27). An irradiance of 5 W/cm$^2$ at 495 nm was chosen to simulate the conditions used in the retinal extraction experiments. Extinction coefficients were determined using the absolute spectra displayed as Fig. S8. The system of ordinary differential equations (ODEs) was solved with a fifth-order Runge-Kutta algorithm written in Python. We neglected in our model a possible photolysis of the thermal decaying states and the Batho intermediate, permitting a bigger step size, namely 250 ns for time constants below 60 $\mu$s for the back reaction and 20 $\mu$s for time constants above 300 $\mu$s. Simulations were run until the equilibrium was reached (convergence). As a measure for accuracy, the mean absolute percentage error was used:

$$accuracy = \frac{100}{3} \times \left( \left| \frac{[Meta_{HPLC}] - [Meta_{Sim}]}{[Meta_{HPLC}]} \right| + \left| \frac{[Rho_{HPLC}] - [Rho_{Sim}]}{[Rho_{HPLC}]} \right| + \left| \frac{[Iso_{HPLC}] - [Iso_{Sim}]}{[Iso_{HPLC}]} \right| \right).$$

## RESULTS

### Large-scale recombinant expression and purification

Wild-type JSR1, tagged at the C-terminus with a 1D4 epitope (3), was stably expressed in suspension cultures of HEK293 GnTI− cells (19). During membrane preparation, JSR1 was reconstituted with 9-*cis* retinal (JSiR1, isorhodopsin) and purified using the 1D4 affinity system followed by SEC (see Methods). Although we also succeeded in reconstituting JSR1 with 11-*cis* retinal (Fig. S1 *A*), we used the 9-*cis* isomer because it leads to the formation of the blue-shifted state isorhodopsin (JSiR1), resulting in the spectral separation of the ground and photoproduct states (Fig. 1). Successful incorporation of the chromophore, sample purity, and concentration were verified by UV-Vis spectroscopy (Figs. 1 and S1). Spectroscopically active and pure JSiR1 elutes as a monodisperse and homogenous sample (Fig. S2) when subjected to size-exclusion chromatography (SEC). Typical yields are about ~0.3 mg per gram of cell mass.

### Features of JSiR1

JSR1 reconstituted with 9-*cis* retinal (JSiR1) exhibits a maximal absorbance in the visible at 505 nm (isorhodopsin, Fig. 1). Irradiation with green light results in a stable hyper- and bathochromic shift of the opsin to an absorption maximum at 535 nm (3). When using 11-*cis* retinal, the ground and photoproduct states have both maximal absorbance at 535 nm (Fig. S1 *A*), as previously shown by Nagata et al. (3). Both the 9-*cis* and 11-*cis* retinals show an hyperchromic shift upon illumination, confirming the bistable nature of the opsin (4). The presence of a Schiff base between retinal and the opsin (Lys 321, JSR1 numbering) was demonstrated using an acid denaturation assay (5). In this assay, opsin molecules are denaturated, and Schiff bases are exposed to solvent, showing absorption maxima at 440 nm (Fig. S1 *B*). To exclude unspecific Schiff bases on possible solvent-exposed lysines, JSiR1 was treated with hydroxylamine ($NH_2OH$) (21). When hydroxylamine cleaves the Schiff base between the retinal and Lys 321, the absorption maxima at 505 nm drops (opsin is formed), and the relative retinal oximes are formed, absorbing around 360 nm (Fig. S3 *A*).

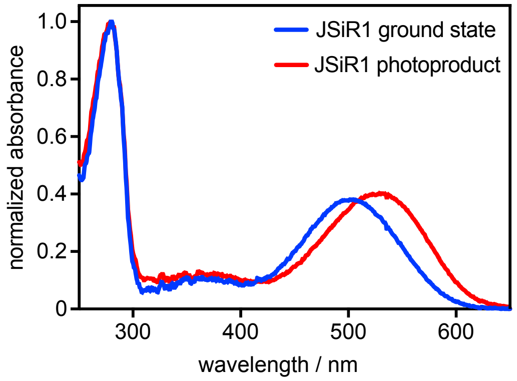


FIGURE 1 UV-Vis spectra of JSiR1. Normalized UV-Vis spectra of JSR1 reconstituted with 9-*cis* retinal (JSiR1, isorhodopsin) and of its photoproduct (Meta) are shown. JSiR1 has maxima absorbance at 505 nm, and JSiR1 photoproduct at 535 nm.

The molar absorption and retinal composition of both JSiR1 ground state and JSR1 photoproduct were then investigated. The extinction coefficient of JSiR1 ground state was calculated assuming that one molecule of JSiR1 releases one molecule of 9-*cis* retinal, and thus $\Delta 360/\Delta 505 = \varepsilon_{retinal}/\varepsilon_{JSiR1}$ (see Methods; (21,22)). JSiR1 ground-state extinction coefficient was found to be 32,660 $M^{-1}$ $cm^{-1}$, where the $\varepsilon_{retinal}$ is 46,930 $M^{-1}$ $cm^{-1}$ (Fig. S4). Fig. S3 *C* shows the correlation between increase/release of 9-*cis* retinal, respectively. The slope of the intercept corresponds to the previous relationship, and its negative value (−1.37 ± 0.05) confirms that when $\Delta 360$ decreases, $\Delta 505$ increases and vice versa.

As for other bistable opsins, the photoproduct of JSR1 is thermally stable and also results from equilibrium of diverse species, depending on parameters such as pH, temperature, and illumination. For the scope of this study, we consider the JSR1 photoproduct (acid-Meta or Meta) as the illuminated form of the receptor at pH 6.5 as described in Methods (6,28). By using the relation $OD_{JSiR1}/\varepsilon_{JSiR1} = OD_{photoproduct}/\varepsilon_{photoproduct}$, the extinction coefficient of

Meta is 37,560 $M^{-1}$ $cm^{-1}$, which is 1.15 times higher than the JSiR1 ground state and similar to the one of squid rhodopsin (22). The slope of intercept in Fig. S5 *B* corresponds to $OD_{photoproduct}/OD_{ground\ state}$ ratio (1.13 $\pm$ 0.04), in other words, to the increment of the extinction coefficient in the photoproduct.

To further characterize chromophore composition in the recombinantly produced JSiR1, we isolated the retinal oximes both from Iso and Meta states (23) and analyzed the relative abundance of their conformations by HPLC. The data (Figs. 2 and S5) reveal an overall homogenous sample before as well as after light irradiation. The photoproduct composition is slightly more heterogeneous as found in other bistable opsins (29,30), likely resembling one of their features.

## Photoreaction dynamics: Time-resolved UV-Vis spectroscopy on JSiR1

Photoactivation of opsins proceeds through a series of intermediates that can be traced by the spectral changes of retinal (31,32). To characterize the spectroscopic intermediates in the photoactivation of JSiR1, we probed transient absorption changes in the UV-Vis range (400–620 nm) across the time regime of 50 ns–900 ms after pulsed excitation. Photoexcitation was accomplished by a 10 ns laser pulse tuned to the emission wavelength of 465 nm to induce the electronic excitation of the chromophore 9-*cis* retinal. This blue-shifted excitation wavelength (relative to the measured absorbance maximum, Fig. 1) was chosen to minimize excitation of the first photointermediate whose short lifetime falls into the pulse width of the laser source. We separately probed the reaction from isorhodopsin to the thermally stable state (i.e., 9-*cis* to all-*trans* retinal isomerization) (Fig. 3 *A*) and the "equilibrium" reaction (Fig. 3 *B*). The latter corresponds to a preilluminated sample consisting of a mixture of the Meta state (with all-*trans* retinal) and the ground state (Rho with 11-*cis* retinal). Illumination of JSiR1 induces the formation of Meta as well as the reaction from Meta to Rho, generating a stable mixture of the latter two states with their concentration ratio being dependent on their lifetimes. Representative time traces at 580, 520, and 480 nm are shown in the top panel of Fig. 3. The data sets were globally fitted using a kinetic model based on a linear

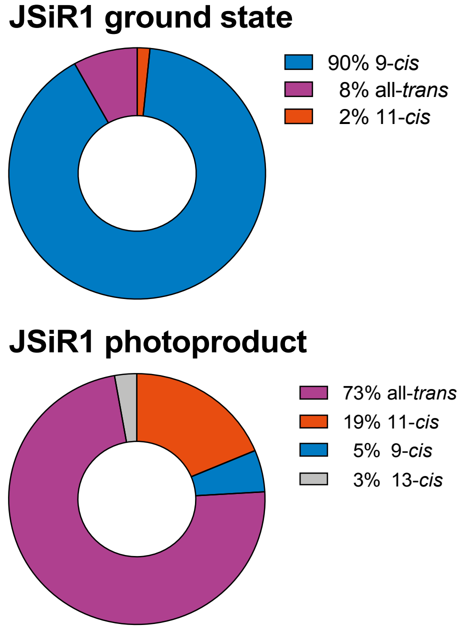


FIGURE 2 HPLC analysis of retinal oximes. Chromophore configurations of JSiR1 before and after light irradiation are shown. Retinal isomers have been grouped and reported in percentages. Raw data are reported in Fig. S6.

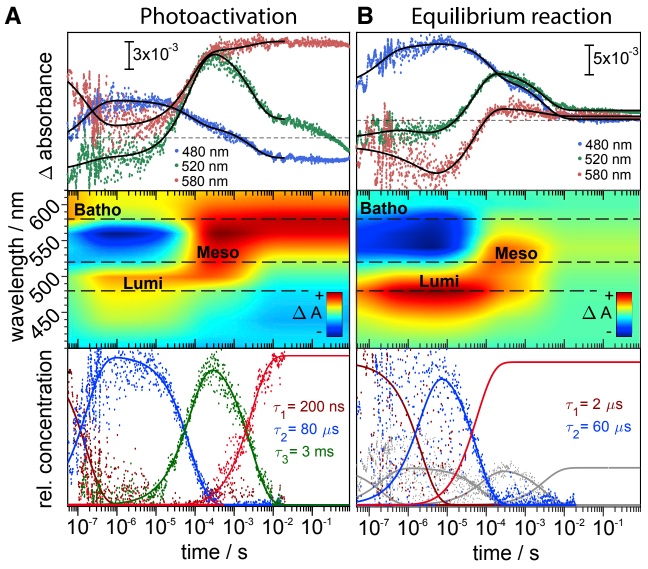


FIGURE 3 Time-resolved UV-Vis absorption changes of JSiR1 at 20°C induced by a short laser flash. (*A*) The left panel refers to the photoreaction of isorhodopsin JSR1 (JSR1 with 9-*cis* retinal), and the right panel (*B*) to the transient absorption changes of an illuminated sample consisting of a Meta-Rho equilibrium. (*A*, *top*) Time traces recorded at three representative wavelengths (480, 520, and 580 nm) are shown. The continuous black line is a global fit to the sum of three exponentials. Zero is indicated as a dashed gray line. (*A*, *middle*) A contour plot of the photoreaction of isorhodopsin with intermediate states as indicated is shown. Data shown are the result of the global fit with a stable final state (Meta). Changes after 20 ms are neglected because of a possible photolysis of the thermally stable Meta state provoked by the measuring light. (*A*, *bottom*) Concentration profiles of the various intermediate states and their associated time constants of decay are shown. Dots represent the contribution of the decay-associated spectra. (*B*, *top*) Time traces recorded at three representative wavelengths (480, 520, and 580 nm) are shown. The continuous black line is the result of a global fit analysis. Zero is indicated as a dashed gray line. (*B*, *middle*) A contour plot of the photoreaction of the Meta-Rho equilibrium with intermediate states as indicated is shown. (*B*, *bottom*) Concentration profile of the used model for the global fit analysis is shown. The sum of two exponentials was fitted with the contribution of the forward reaction held constant (*gray*; same as in *A*, *bottom*). The amplitude difference serves just as an accentuation of the Meta-to-Rho reaction and does not reflect actual concentrations.

sequence of unidirectional reactions (see *bottom panels* of Fig. 3, *A* and *B* for the fitted concentration profiles).

A series of three sequential transitions is involved in the transition from Iso to the stable state (Fig. 3 *A*, *middle* and *bottom panels*). The resulting time constants obtained for each transition are $\tau_1 = 200$ ns, $\tau_2 = 80$ $\mu$s, and $\tau_3 =$ 3 ms. It has been shown for bovine rhodopsin that Iso and Rho share a common Batho intermediate in the forward photoreaction and subsequently follow the same reaction path (33). On that account, we consider the Iso-to-Meta reaction to consist of the same intermediate states as the Rho-to-Meta transition. An early intermediate with an absorption difference maximum at 600 nm rises beyond the time resolution of our experiment (50 ns). We termed this early red-shifted intermediate Batho in accordance with the photoactivation cycle of other bistable opsins (octopus and squid rhodopsin (26,27)). Formation of the intermediate states is accompanied by the depletion of the initial state as detected by the negative absorption at around 550 nm at early timescale. The decay of Batho, with a time constant of 200 ns, results in an absorbance increase at around 480 nm, indicating the formation of a new early blue-shifted Lumi intermediate (*blue trace* in Fig. 3 *A*, *middle panel*). Although the Lumi intermediates in squid and octopus rhodopsin are red-shifted with respect to the 11-*cis* state Rho (8,32), it is blue-shifted in JSR1. Lumi decays with $\tau_2 = 80$ $\mu$s and leads to the formation of an intermediate with an absorption difference maximum at 520 nm called Meso (*green trace* in Fig. 3 *A*, *middle panel*). At the later stage, the absorption change decreases at around 520 nm, whereas a slight increase is observed at around 580 nm. At this time point, the thermally stable Meta state is formed after pulsed excitation, as inferred from the similarity to the steady-state difference spectrum of Meta-minus-Iso (Fig. S7 *A*). The observed subsequent changes could originate from a precursor to the final Meta state, as has been suggested for octopus rhodopsin (34). In this case, the photoreaction would be considerably slower than that of any other reaction of mono- or bistable opsins. However, it may well be that the thermally stable Meta is excited by the probing light, as observed for the long-lived P4 state of channelrhodopsin-2 (35). Omitting the changes that occur after 20 ms, the time constant for the decay of Meso is 3 ms, similar to squid rhodopsin (36).

In bistable opsins, illumination of the Meta state leads to isomerization of all-*trans* retinal to the 11-*cis* configuration corresponding to the native Rho state (11,12). We characterized this process by generating a Meta-Rho equilibrium by illumination of the initial JSiR1 state with an intense laser emitting at 473 nm. The photoreaction of the Meta and Rho states was initiated by applying pulsed laser excitation. The time-resolved absorbance changes are depicted in Fig. 3 *B*. The photoreaction of the preilluminated sample also involves Batho (600 nm), Lumi (480 nm), and Meso (510 nm) states, reasoning for the same reaction path of Iso and Rho. It is evident that the light-induced absorption changes of the Meta-Rho mixture (Fig. 3 *B*) exhibit distinct differences as compared to the Iso-to-Meta reaction (Fig. 3 *A*). The most striking deviation is the absence of a strong positive absorption change at the end of the data recording, which indicates that the interconversion of Meta and Rho does not result in any change of absorbance. This fact reflects the similar absorption characteristics of Meta and Rho (Fig. S1). The Meta-Rho depletion, represented by the early negative absorption change, is red-shifted compared to the Iso-to-Meta reaction, representing the spectral red shift from Iso (505 nm) to Rho/Meta (535 nm). Due to this shift, Meso decreases in absorbance, and Batho is barely visible. In contrast, Lumi appears more pronounced. The rise of the time trace at 480 nm, characteristic for Lumi, clearly shows biphasic behavior that is even more pronounced in the ground-state depletion represented by the kinetic trace at 520 nm (Fig. 3 *B*, *top*). Because the laser flash excites both the Meta and the Rho states, these photoreactions are overlaid, leading to the biphasic behavior. Meso, on the other hand, forms and decays in a monoexponential way.

To disentangle forward and reverse reactions, we searched for kinetic signatures that emerge on top of those of the activation process. Global analysis revealed that two additional transitions with time constants of 2 and 60 $\mu$s were necessary to fit the data, taking account of the biphasic rise and decay of Lumi (Fig. 3 *B*, *bottom panel*). This observation is indicative of a photoreverse reaction consisting of two intermediate states, further referred to as $M_1$ and $M_2$. Although the proposed recovery reaction of octopus rhodopsin involves two intermediates and is considerably slower than the activation process (11), the opposite is true for JSR1. It has been proposed for squid rhodopsin that the retinal Schiff base de- and reprotonates during the regeneration of Rho, indicated by an intermediate with an absorption maximum of 380 nm (12). We have not observed such a blue-shifted intermediate in the photoreaction of JSR1, which argues against transient deprotonation of the retinal Schiff base during the Meta-to-Rho reaction. This conclusion is in agreement with our resonance Raman experiments (vide infra).

Based on our time-resolved experiments, we propose a model of the two-photon cycle of JSR1 (Fig. 4). The absorption maxima of the intermediates were determined by calculating absolute spectra (Fig. S8) from the difference absorption experiments. In the next step, we validated this model by a kinetic simulation that should reproduce the relative contributions of the intermediate states as determined by retinal extraction and HPLC analysis (Fig. 2). The reactions were simulated by a system of coupled ODEs excluding photoreactions of the thermal states. These ODEs depend on the irradiance, the quantum efficiencies of the intermediate states (Iso, Rho, and Meta), and their extinction coefficients (see Methods for details). Translating

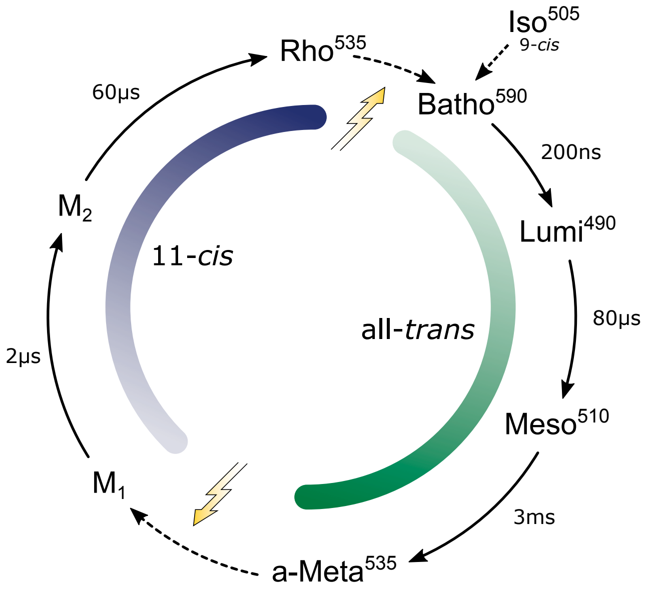


FIGURE 4 Proposed reaction scheme of JSR1. A first photon triggers Iso and/or Rho isomerization as part of the forward reaction (all-*trans* half-cycle, in *green*). A second photon stimulates Meta and initiates the backward reaction (11-*cis* half-cycle, in *blue*). Iso, Rho, and Meta are thermally stable states that are able to start a photoreaction upon absorption of a photon. Time constants and absorption maxima are derived from the flash photolysis data (Fig. 3).

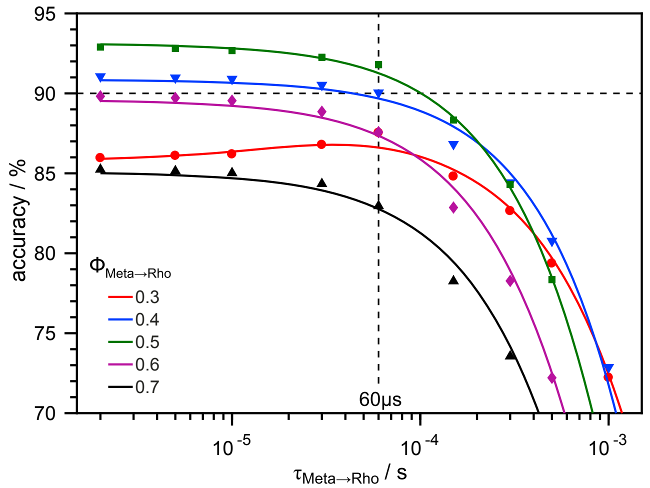


FIGURE 5 Photostationary state simulation. Accuracy for different simulation scenarios assumes $\Phi_{Rho} = 0.7$ and a quantum efficiency of 0.06 for the Meta-to-Iso transition. Best results are obtained for $\Phi_{Meta} =$ 0.4–0.5 and for time constants of less than 60 $\mu$s for the reverse reaction from Meta to Rho.

the photoconversion of 6–9% of Iso in the time-resolved experiment yields a quantum efficiency of 0.2–0.3 for the Iso-to-Meta transition, which is comparable to other mono- and bistable opsins (for the simulation, a value of 0.25 was used) (22,37) (for a detailed analysis, see Supporting Materials and Methods). An accuracy above 90% is considered to be in accordance with the HPLC data because of the experimental error in the determination of the retinal isomers. Fig. 5 shows the accuracy of the different scenarios, assuming a typical quantum efficiency of 0.7 for Rho (22,37). For time constants below 10 $\mu$s, the formation of Meta is so fast that its population depends only on the Meta-to-Rho quantum efficiency. Above 10 $\mu$s, the duration of the reverse reaction starts to take influence, decreasing drastically the overall accuracy. This indicates that Meta can only accumulate after continuous illumination if the reverse reaction is considerably faster than the photoactivation process. Best results are achieved with Meta-to-Rho quantum efficiencies of 0.4 and 0.5 and time constants below 60 $\mu$s, supporting the proposed photocycle (Fig. 4).

## Structural changes associated with photoswitching: Vibrational spectroscopy on JSiR1

Resonance Raman spectroscopy is a technique that selectively enhances vibrations of the retinal. Under continuous illumination, JSiR1 forms a photostationary mixture consisting of Rho and Meta as well as other intermediate states. To reduce their contribution in a Raman spectrum, we used a preilluminated JSiR1 solution and a rotational cuvette always providing a sample in the laser spot where all the intermediates have decayed. The top red spectrum in Fig. 6, recorded with a probing wavelength of 532 nm, shows a C=C stretch peak at 1536 $cm^{-1}$ that matches the correlation to the electronic absorption appearing in the UV-Vis range (Fig. 1) (38). The fingerprint region (1100–1400 $cm^{-1}$) exhibits bands that are mostly due to C-C stretching modes coupled to C-C-H bends and is very sensitive to the isomeric state of the retinal (33). The peak pattern at around 1201 $cm^{-1}$ bears great similarity to the spectrum of the (light-adapted) ground-state of bacteriorhodopsin (39), i.e., all-*trans* is the predominant configuration, which is characteristic for the Meta state of JSR1. However, hydrogen-out-of-plane (HOOP) modes are usually weak for all-*trans* Meta states, whereas they are more pronounced in Rho, reasoning for a distortion of the polyene chain in the 11-*cis* configuration (34,40,41). Based on this, the intense HOOP band at 967 $cm^{-1}$ is partially a result of the presence of Rho in our spectrum. The highest frequency band in a resonance Raman spectrum of any rhodopsin is the C=N-H vibration of the retinal Schiff base. The band at 1644 $cm^{-1}$ downshifts by 22 $cm^{-1}$ upon deuteration and, hence, is clearly assigned to a protonated Schiff base vibration, here of both Meta and Rho (Fig. 6, *inset*).

For recording the spectrum of isorhodopsin JSiR1, a sample was cooled to −193°C while keeping it strictly in the dark (*middle green spectrum*). The thermal energy is so low under these conditions that only the Batho intermediate is formed after light excitation. It has been shown for bovine, octopus, and squid rhodopsin that the HOOP modes of retinal appearing at around 950 $cm^{-1}$ are characteristic

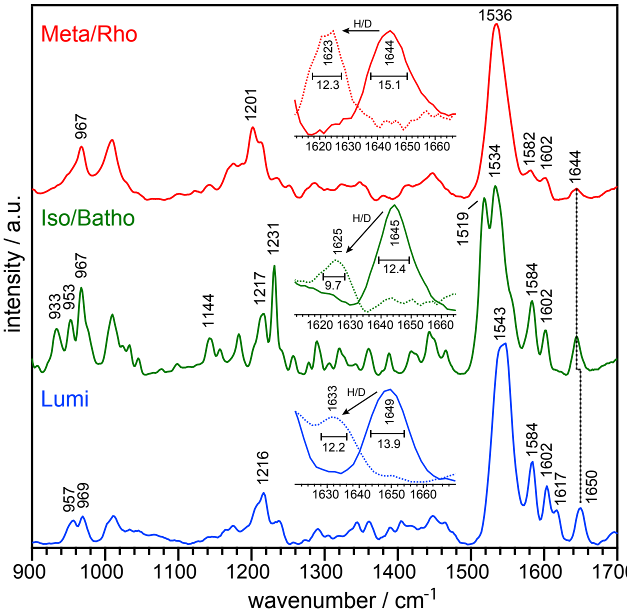


FIGURE 6 Resonance Raman spectroscopy on JSiR1. Top spectrum refers to a Meta-Rho mixture recorded with a rotational cuvette under resonant conditions (Raman probe at 532 nm). The band pattern in the fingerprint region is characteristic for an all-*trans* retinal, pointing to a main contribution of Meta. The C=N-H vibration of the retinal Schiff base was identified via H/D exchange (*inset*). Middle spectrum was taken at 80 K (−193°C). HOOP modes and C=C stretch vibrations are bands attributed to isorhodopsin and the Batho intermediate. Fingerprint region shows characteristic peaks for a 9-*cis* configuration. Schiff base frequency is the same as in Meta-Rho (*inset*). Bottom spectrum was taken at 183 K (−90°C) using a pump/probe scheme with 532/457 nm lasers, respectively. The C=C stretch vibration at 1543 $cm^{-1}$ correlates with the visible absorption maximum of 490 nm of Lumi. Schiff base vibration is shifted to a higher frequency (*inset*).

for different states (8,42,43). On this basis, the band at 953 $cm^{-1}$ can be assigned to isorhodopsin. Also, the fingerprint band pattern with peaks at 1144, 1217, and 1231 $cm^{-1}$ shows similarity to a 9-*cis* configured retinal (44). The ethylenic region exhibits two prominent peaks at 1519 and 1534 $cm^{-1}$. Both frequencies are not correlated to the electronic absorption maximum of Iso at 505 nm. The band at 1519 $cm^{-1}$ translates to a more red-shifted absorption maximum, possibly from Batho. This conclusion is supported by the HOOP mode at 933 $cm^{-1}$, which is at 940 $cm^{-1}$ for Batho in octopus and squid rhodopsin (8,44). The highest frequency mode is with 1644 $cm^{-1}$ at the same position as in Rho, and its deuterium isotope shift is 19 $cm^{-1}$ (Fig. 6 *inset*). There are no further bands, which indicates that if Batho is spectroscopically present, its C=N frequency is the same as in Rho and Iso. This means that the Schiff base environment is identical for Iso, Rho, Batho, and Meta, which was also found for octopus and squid rhodopsin (8,44).

By increasing the temperature, thermal transitions after Batho can take place. For octopus rhodopsin, the Lumi intermediate is stable between −115 and −65°C (45). The bottom blue spectrum in Fig. 7 was recorded at −90°C. A Raman probe wavelength of 457 nm was chosen for selective resonance enhancement of the blue-shifted Lumi intermediate. In addition, a laser with 532 nm was used to initiate the photoconversion from Iso to Lumi. The spectrum shows a strong C=C band at 1543 $cm^{-1}$, which matches the electronic absorption maximum of Lumi at 490 nm. Another characteristic feature of Lumi is the HOOP mode at 957 $cm^{-1}$ (42,46). There is a small shoulder at 1617 and a distinct peak at 1650 $cm^{-1}$ in the Schiff base region above 1600 $cm^{-1}$. Upon $H_2O/D_2O$ exchange, the latter band shifts to 1630 $cm^{-1}$, indicating that the Schiff base is also protonated in Lumi (Fig. 6 *inset*). Interestingly, the C=N stretching frequency in $D_2O$ is higher in Lumi than compared to the other states, arguing for an increase of the force constant of this bond. This is contrary to the behavior in octopus rhodopsin (47,48).

By analyzing the bandwidths of the C=N-H vibrational bands of the retinal Schiff base, it is possible to identify the existence of a neighboring water molecule (49). Resonance energy transfer between the Schiff base vibration and the bending mode of $H_2O$ is broken upon exchange of $H_2O$ for $D_2O$. This leads to a narrowing of the Schiff base band upon H/D exchange, which can be observed for the Meta-Rho (from 15.1 ± 1.0 $cm^{-1}$ in $H_2O$ to 12.3 ± 1.8 $cm^{-1}$ in $D_2O$) and Iso/Batho (from 12.4 ± 0.3 $cm^{-1}$ in $H_2O$ to 9.7 ± 2.5 $cm^{-1}$ in $D_2O$) spectra in Fig. 6. This suggests a water molecule in close vicinity to the Schiff

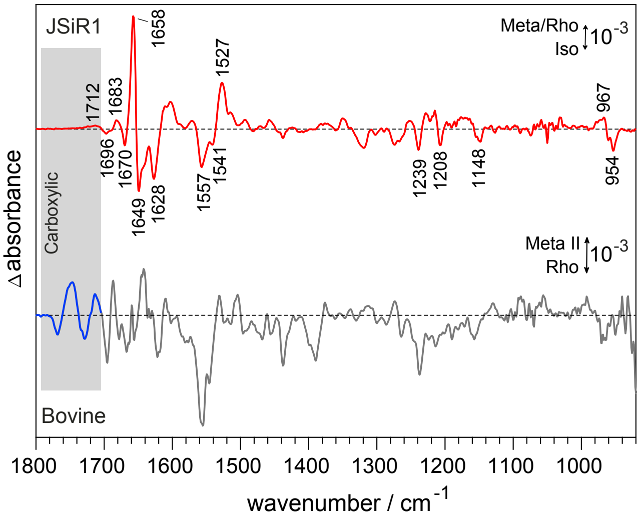


FIGURE 7 Light-induced FTIR absorption difference spectrum of JSiR1. The top spectrum in red reflects the vibrational changes that take place upon illumination by an LED emitting at 470 nm of Iso (negative bands) to a Meta-Rho mixture (positive bands). The frequencies of some of the difference bands are indicated and discussed in the text. The absence of pronounced bands in the carboxylic region above 1700 $cm^{-1}$ is in contrast to the photoactivation of bovine rhodopsin (*bottom spectrum*) in which strong carboxylic bands arise. Formation of Meta II is accompanied by strong carboxylic bands as a consequence of the deprotonation of the Schiff base.

base in these states. This effect is less pronounced for the Lumi state, but the analysis is more error prone (13.9 ± 0.5 $cm^{-1}$ in $H_2O$ to 12.2 ± 3.7 $cm^{-1}$ in $D_2O$). Thus, we cannot unequivocally conclude on the presence of the water molecule close to the Schiff base in the Lumi state. However, the similar deuterium isotope shift in all investigated states, namely Iso, Rho, Meta, Batho, and Lumi, implies that the hydrogen bond strength of the Schiff base to the water part of the counterion system remains constant (from JSiR1 structure, unpublished data).

Although resonance Raman spectroscopy focuses on the retinal chromophore, infrared spectroscopy reveals structural information of the entire protein. We performed light-induced FTIR difference spectroscopy on the 9-*cis* bound JSiR1 to investigate the structural rearrangements in the protein backbone and protonation changes of amino acids as well as conformational changes of the retinal upon light activation (50,51). In the difference spectrum (Fig. 7, *red spectrum*), negative bands (−) correspond to vibrations in isorhodopsin (dark state) and positive bands (+) to the Meta-Rho mixture (illuminated state).

Some of the bands can be assigned to the retinal chromophore. The 1559(−),1541(−)/1527(+) $cm^{-1}$ feature is attributed to the C=C stretch of Iso and Meta-Rho, respectively. This assignment is aided by comparison to the differences in the Raman spectra of Meta-Rho and Isorhodopsin (Fig. S9). A negative band at 1519 $cm^{-1}$ is missing in the FTIR difference spectrum, arguing for its attribution to Batho in the Raman spectrum (Fig. 6). In the fingerprint region, the three negative bands at 1148, 1208, and 1239 $cm^{-1}$ are characteristic for the 9-*cis* configuration of Iso (52). The negative HOOP mode at 954 $cm^{-1}$ can be attributed to Iso, whereas the positive feature at 967 $cm^{-1}$ indicates that Rho is also formed in addition to Meta, as shown for octopus rhodopsin (42). The most intense difference bands can be found in the region between 1600 and 1700 $cm^{-1}$ containing mostly amide I (C=O stretching) vibrations. These originate from the protein backbone and thus reflect structural rearrangements. Contributions from the C=N-H vibration of the retinal Schiff base can be ruled out from the comparison with our Raman measurements (Fig. 6), which showed that the C=N-H stretching frequency is identical for the Rho, Iso, and Meta states. Thus, the corresponding vibrational bands cancel in the FTIR difference spectrum of these intermediates (8,42).

The positive bands in the FTIR spectrum, corresponding to the Meta and Rho states, do not show evident similarities to published spectra of bovine and octopus rhodopsin (42,43). This is expected because the difference spectrum reflects the vibrational changes corresponding to the 9-*cis* to all-*trans*/11-*cis* transition (Iso-to-Meta-Rho) for JSR1 and not to an 11-*cis* to all-*trans* (Rho-to-Meta) as for visual rhodopsin. It was found for bovine and octopus rhodopsin that Rho and Iso predominately differ in the retinal moiety and thus an infrared difference spectrum does not exhibit strong bands above 1600 $cm^{-1}$ (42,53). This suggests that the pronounced changes in amide I vibrations are linked to the Iso-to-Meta transition, meaning that these correspond to structural changes of the opsin moiety upon photoactivation.

Hydrogen-bonding changes and protonation reactions of carboxylic acid residues can be monitored in the region above 1700 $cm^{-1}$. JSiR1 does not display any distinct bands in this region, except for a weak feature at 1712 $cm^{-1}$. This observation is reminiscent to bovine rhodopsin, in which Meta I does not show pronounced bands in this region. Yet, strong difference bands appear in the signaling state Meta II because of the deprotonation of the Schiff base (Fig. 7, *black spectrum*). A similar behavior to JSiR1 was observed in the Meta state of octopus rhodopsin (42,54). Taken together, our spectroscopic results strongly suggest that the Schiff base remains protonated throughout the two-photon cycle of JSR1.

## DISCUSSION

The inactive and active states (Rho and Meta) of JSR1 have similar absorption characteristics, making spectroscopic measurements a hard-methodical challenge. Reconstitution of JSR1 with 9-*cis* retinal resulted in the formation of the blue-shifted inactive isorhodopsin (JSiR1, Fig. 1), which permits the spectroscopic discrimination of ground and photoproduct states. We succeeded at producing highly homogenous samples of JSiR1 for time-resolved experiments. Illumination of the isorhodopsin form of JSR1 results in the formation of the thermally stable Meta state within 3 ms. Transition to this state involves three intermediates, namely Batho (590 nm), Lumi (490 nm), and Meso (510 nm). The photorecovery consists of at least two intermediates and is considerably faster than the activation process, which is confirmed by photostationary state simulations. Although we have kinetic data only for the Iso-to-Meta transition, it can be concluded from the measurements on the illuminated JSiR1 that Rho follows the same reaction path as Iso, as observed in other opsins (8,33). A unique feature of JSR1 is the relatively large blue shift of the Lumi intermediate. However, deprotonation of the Schiff base does not take place at this stage because such an event would shift the absorption maximum far lower, as seen in other rhodopsins (e.g., bovine rhodopsin, octopus rhodopsin, or bacteriorhodopsin (31,32,38)) and in JSR1 deprotonated mutants (unpublished data). This conclusion is solidified by resonance Raman spectroscopy on the Lumi state, in which the C=N-H vibration of the retinal Schiff base exhibits a deuterium isotope effect, implying the presence of a proton. Based on these initial experiments, we propose a photocycle model of the two-photon reaction of JSR1 as illustrated in Fig. 4.

A quantum efficiency of around 0.7 for Rho is common among bistable opsins, whereas it is 0.4–0.5 for Meta

(22,37). Our photostationary state simulations show that the lower quantum efficiency in concert with a faster photorecovery ensures that Meta accumulates after illumination. Using our derived reaction scheme of JSR1 (Fig. 4), we were able to quantitatively reproduce the ratio of isomeric states determined by retinal extraction and HPLC analysis, providing additional support for our photocycle model. Assuming that the quantum efficiency of Rho in JSR1 is also 0.7, our simulations suggest that the quantum efficiency for the photorecovery is also between 0.4 and 0.5.

Light-induced FTIR difference spectroscopy revealed structural insights into the mechanism of photoactivation. The formation of Meta is accompanied by structural changes in the protein backbone, as is the case in bovine rhodopsin upon its conversion to metarhodopsin II, which is the state able to activate the G-protein (55). Spectroscopically, the change of the latter state can be identified by following the deprotonation of the Schiff base, resulting in a distinct blue shift of the electronic transition from 498 to 380 nm (31). However, the Meta state of JSR1 neither exhibits such a shift nor shows carboxylic bands in the FTIR difference spectrum equivalent to metarhodopsin II (43). Furthermore, our resonance Raman experiments have shown that retinal Schiff base is protonated in the Meta state. The similar shift upon deuteration in the inactive and active states indicates that the hydrogen bond of the Schiff base proton to the water of the counterion system (observed in the crystal structure of JSiR1, unpublished data) (56) remains constant. A similar behavior is found in octopus rhodopsin, another invertebrate rhodopsin in which the Schiff base is also reported not to deprotonate (42,54). The band at 1712 $cm^{-1}$ is therefore due to a protonated carboxylic group residing in different environments in the Iso and Meta states, such as Asp147 in the DRY motif at the cytoplasmic end of transmembrane helix VI (57). Our results strongly favor a scenario in which retinal isomerization induces structural changes in JSR1, eventually leading to the formation of Meta and then G-protein activation but without requiring deprotonation of the Schiff base.

We believe that our spectroscopic characterization of the two-photon cycle of JSiR1 forms the basis for future ultrafast spectroscopic and structural studies on bistable opsins. High expression yields and room temperature stability identify JSR1 as an excellent sample for time-resolved serial crystallographic studies at synchrotrons and x-ray free electron lasers (58,59). Thus, we expect that JSR1 may serve as a model protein for further our understanding of the molecular basis of bistability in light-sensitive GPCRs and for designing new light-sensitive switches for optogenetic applications.

## SUPPORTING MATERIAL

Supporting Material can be found online at https://doi.org/10.1016/j.bpj.2019.02.025.

## AUTHORS CONTRIBUTIONS

G.F.X.S., J.H., and E.L. conceived the study. N.V., M.K., and E.L. cloned, expressed, and purified JSR1. They also performed the biochemical analysis of the sample and relative calculations. D.E. performed time-resolved UV-Vis, FTIR, and Raman spectroscopic experiments, photostationary state simulations, and the global fit analysis. All the authors contributed to data analysis, interpretation, and writing the manuscript.

## ACKNOWLEDGMENTS

Petr Skopintsev is acknowledged for his contribution to data analysis, and Björn Süss for kindly providing the Meta II bovine difference spectrum.

This project was supported by the following agencies: Human Frontier Science Program Research Grant RGP0034/2014, X-Probe ITN 637295, Swiss National Science Foundation 310030B_17333, National Centres of Competence in Research Molecular Systems Engineering to G.F.X.S, Peter und Traudl Engelhorn Stiftung to E.L. and the German Research Foundation (DFG) through SFB-1078, project B3 and SPP-1926, HE 2063/6-1 to J.H.

## REFERENCES

1. Ernst, O. P., D. T. Lodowski, …, H. Kandori. 2014. Microbial and animal rhodopsins: structures, functions, and molecular mechanisms. *Chem. Rev.* 114:126–163.
2. Palczewski, K. 2006. G protein-coupled receptor rhodopsin. *Annu. Rev. Biochem.* 75:743–767.
3. Nagata, T., M. Koyanagi, …, A. Terakita. 2012. Depth perception from image defocus in a jumping spider. *Science.* 335:469–471.
4. Tsukamoto, H., and A. Terakita. 2010. Diversity and functional properties of bistable pigments. *Photochem. Photobiol. Sci.* 9:1435–1443.
5. Matsuyama, T., T. Yamashita, …, Y. Shichida. 2012. Photochemical properties of mammalian melanopsin. *Biochemistry.* 51:5454–5462.
6. Hubbard, R., and R. C. St George. 1958. The rhodopsin system of the squid. *J. Gen. Physiol.* 41:501–528.
7. Koyanagi, M., and A. Terakita. 2014. Diversity of animal opsin-based pigments and their optogenetic potential. *Biochim. Biophys. Acta.* 1837:710–716.
8. Sulkes, M., A. Lewis, and M. A. Marcus. 1978. Resonance Raman spectroscopy of squid and bovine visual pigments: the primary photochemistry in visual transduction. *Biochemistry.* 17:4712–4722.
9. Nishioku, Y., M. Nakagawa, …, M. Terazima. 2002. Energetics and volume changes of the intermediates in the photolysis of octopus rhodopsin at a physiological temperature. *Biophys. J.* 83:1136–1146.
10. Nakagawa, M., S. Kikkawa, …, M. Tsuda. 1997. Light-induced protein conformational changes in the photolysis of octopus rhodopsin. *Biophys. J.* 72:2320–2328.
11. Inoue, K., M. Tsuda, and M. Terazima. 2007. Photoreverse reaction dynamics of octopus rhodopsin. *Biophys. J.* 92:3643–3651.
12. Tsuda, M. 1978. Kinetic study of photoregeneration process of digitonin-solubilized squid rhodopsin. *Biochim. Biophys. Acta.* 502:495–506.
13. Shirzad-Wasei, N., and W. J. DeGrip. 2016. Heterologous expression of melanopsin: present, problems and prospects. *Prog. Retin. Eye Res.* 52:1–21.
14. Murakami, M., R. Kitahara, …, T. Kouyama. 2007. Crystallization and crystal properties of squid rhodopsin. *Acta Crystallogr. Sect. F Struct. Biol. Cryst. Commun.* 63:475–479.
15. Murakami, M., and T. Kouyama. 2008. Crystal structure of squid rhodopsin. *Nature.* 453:363–367.
16. Schertler, G. F. 2008. Signal transduction: the rhodopsin story continued. *Nature.* 453:292–293.

17. Shimamura, T., K. Hiraki, …, M. Miyano. 2008. Crystal structure of squid rhodopsin with intracellularly extended cytoplasmic region. *J. Biol. Chem.* 283:17753–17756.

18. Koyanagi, M., T. Nagata, …, F. Tokunaga. 2008. Molecular evolution of arthropod color vision deduced from multiple opsin genes of jumping spiders. *J. Mol. Evol.* 66:130–137.

19. Reeves, P. J., N. Callewaert, …, H. G. Khorana. 2002. Structure and function in rhodopsin: high-level expression of rhodopsin with restricted and homogeneous N-glycosylation by a tetracycline-inducible N-acetylglucosaminyltransferase I-negative HEK293S stable mammalian cell line. *Proc. Natl. Acad. Sci. USA.* 99:13419–13424.

20. Kito, Y., T. Suzuki, …, Y. Sekoguti. 1968. Absorption spectrum of rhodopsin denatured with acid. *Nature.* 218:955–957.

21. Wald, G., and P. K. Brown. 1953. The molar extinction of rhodopsin. *J. Gen. Physiol.* 37:189–200.

22. Suzuki, T., K. Uji, and Y. Kito. 1976. Studies on cephalopod rhodopsin: photoisomerization of the chromophore. *Biochim. Biophys. Acta.* 428:321–338.

23. Groenendijk, G. W., W. J. de Grip, and F. J. Daemen. 1979. Identification and characterization of syn- and anti-isomers of retinaloximes. *Anal. Biochem.* 99:304–310.

24. Trehan, A., R. S. H. Liu, …, T. Yoshizawa. 1990. On retention of chromophore configuration of rhodopsin isomers derived from three dicis retinal isomers. *Bioorg. Chem.* 18:30–40.

25. Lórenz-Fonfría, V. A., T. Resler, …, J. Heberle. 2013. Transient protonation changes in channelrhodopsin-2 and their relevance to channel gating. *Proc. Natl. Acad. Sci. USA.* 110:E1273–E1281.

26. Radu, I., C. Bamann, …, J. Heberle. 2009. Conformational changes of channelrhodopsin-2. *J. Am. Chem. Soc.* 131:7313–7319.

27. Strickler, S. J., and R. A. Berg. 1962. Relationship between absorption intensity and fluorescence lifetime of molecules. *J. Chem. Phys.* 37:814–822.

28. Yoshizawa, T. 1972. The behaviour of visual pigments at low temperatures. *In* Photochemistry of Vision, Handbook of Sensory Physiology. H. J. A. Dartnall, ed. Springer, pp. 146–179.

29. Sakai, K., T. Yamashita, …, Y. Shichida. 2015. Diversity of active states in TMT opsins. *PLoS One.* 10:e0141238.

30. Kojima, K., T. Yamashita, …, Y. Shichida. 2017. Evolutionary steps involving counterion displacement in a tunicate opsin. *Proc. Natl. Acad. Sci. USA.* 114:6028–6033.

31. Kliger, D. S., and J. W. Lewis. 1995. Spectral and kinetic characterization of visual pigment photointermediates. *Isr. J. Chem.* 35:289–307.

32. Nishioku, Y., M. Nakagawa, …, M. Terazima. 2001. A spectrally silent transformation in the photolysis of octopus rhodopsin: a protein conformational change without any accompanying change of the chromophore's absorption. *Biophys. J.* 80:2922–2927.

33. Kliger, D. S., J. S. Horwitz, …, C. M. Einterz. 1984. Evidence for a common BATHO-intermediate in the bleaching of rhodopsin and isorhodopsin. *Vision Res.* 24:1465–1470.

34. Doukas, A. G., B. Aton, …, T. G. Ebrey. 1978. Resonance Raman studies of bovine metarhodopsin I and metarhodopsin II. *Biochemistry.* 17:2430–2435.

35. Resler, T., B. J. Schultz, …, J. Heberle. 2015. Kinetic and vibrational isotope effects of proton transfer reactions in channelrhodopsin-2. *Biophys. J.* 109:287–297.

36. Shichida, Y., and H. Imai. 1998. Visual pigment: G-protein-coupled receptor for light signals. *Cell. Mol. Life Sci.* 54:1299–1315.

37. Suzuki, T., and R. H. Callender. 1981. Primary photochemistry and photoisomerization of retinal at 77 degrees K in cattle and squid rhodopsins. *Biophys. J.* 34:261–270.

38. Althaus, T., W. Eisfeld, …, M. Stockburger. 1995. Application of Raman spectroscopy to retinal proteins. *Isr. J. Chem.* 35:227–251.

39. Smith, S. O., J. Lugtenburg, and R. A. Mathies. 1985. Determination of retinal chromophore structure in bacteriorhodopsin with resonance Raman spectroscopy. *J. Membr. Biol.* 85:95–109.

40. Kitagawa, T., and M. Tsuda. 1980. Resonance Raman spectra of octopus acid and alkaline metarhodopsins. *Biochim. Biophys. Acta.* 624:211–217.

41. Pande, C., A. Pande, …, M. Tsuda. 1987. Resonance Raman spectroscopy of octopus rhodopsin and its photoproducts. *Biochemistry.* 26:4941–4947.

42. Nishimura, S., H. Kandori, …, A. Maeda. 1997. Structural dynamics of water and the peptide backbone around the Schiff base associated with the light-activated process of octopus rhodopsin. *Biochemistry.* 36:864–870.

43. Siebert, F. 1995. Application of FTIR spectroscopy to the investigation of dark structures and photoreactions of visual pigments. *Isr. J. Chem.* 35:309–323.

44. Huang, L., H. Deng, …, R. H. Callender. 1996. A resonance Raman study of the C=N configurations of octopus rhodopsin, bathorhodopsin, and isorhodopsin. *Biochemistry.* 35:8504–8510.

45. Cooper, A., S. F. Dixon, and M. Tsuda. 1986. Photoenergetics of octopus rhodopsin. Convergent evolution of biological photon counters? *Eur. Biophys. J.* 13:195–201.

46. Pan, D., and R. A. Mathies. 2001. Chromophore structure in lumirhodopsin and metarhodopsin I by time-resolved resonance Raman microchip spectroscopy. *Biochemistry.* 40:7929–7936.

47. Masuda, S., E. H. Morita, …, M. Tsuda. 1993. Infrared studies of octopus rhodopsin and lumirhodopsin. *J. Mol. Struct.* 297:29–34.

48. Furutani, Y., Y. Shichida, and H. Kandori. 2003. Structural changes of water molecules during the photoactivation processes in bovine rhodopsin. *Biochemistry.* 42:9619–9625.

49. Hildebrandt, P., and M. Stockburger. 1984. Role of water in bacteriorhodopsin's chromophore: resonance Raman study. *Biochemistry.* 23:5539–5548.

50. Bartl, F. J., and R. Vogel. 2007. Structural and functional properties of metarhodopsin III: recent spectroscopic studies on deactivation pathways of rhodopsin. *Phys. Chem. Chem. Phys.* 9:1648–1658.

51. Radu, I., M. Schleeger, …, J. Heberle. 2009. Time-resolved methods in biophysics. 10. Time-resolved FT-IR difference spectroscopy and the application to membrane proteins. *Photochem. Photobiol. Sci.* 8:1517–1528.

52. Bagley, K. A., L. Eisenstein, …, M. Tsuda. 1989. A comparative study of the infrared difference spectra for octopus and bovine rhodopsins and their bathorhodopsin photointermediates. *Biochemistry.* 28:3366–3373.

53. Kandori, H., and A. Maeda. 1995. FTIR spectroscopy reveals microscopic structural changes of the protein around the rhodopsin chromophore upon photoisomerization. *Biochemistry.* 34:14220–14229.

54. Masuda, S., E. H. Morita, …, M. Tsuda. 1993. Infrared studies of octopus rhodopsin. Existence of a long-lived intermediate and the states of the carboxylic group of Asp-81 in rhodopsin and its photoproducts. *FEBS Lett.* 317:223–227.

55. Hofmann, K. P., P. Scheerer, …, O. P. Ernst. 2009. A G protein-coupled receptor at work: the rhodopsin model. *Trends Biochem. Sci.* 34:540–552.

56. Terakita, A., M. Koyanagi, …, Y. Shichida. 2004. Counterion displacement in the molecular evolution of the rhodopsin family. *Nat. Struct. Mol. Biol.* 11:284–289.

57. Mahalingam, M., K. Martínez-Mayorga, …, R. Vogel. 2008. Two protonation switches control rhodopsin activation in membranes. *Proc. Natl. Acad. Sci. USA.* 105:17795–17800.

58. Panneels, V., W. Wu, …, G. Schertler. 2015. Time-resolved structural studies with serial crystallography: a new light on retinal proteins. *Struct. Dyn.* 2:041718.

59. Neutze, R., and K. Moffat. 2012. Time-resolved structural studies at synchrotrons and X-ray free electron lasers: opportunities and challenges. *Curr. Opin. Struct. Biol.* 22:651–659.

Biophysical Journal, Volume *116*

## Supplemental Information

# The Two-Photon Reversible Reaction of the Bistable Jumping Spider Rhodopsin-1

**David Ehrenberg, Niranjan Varma, Xavier Deupi, Mitsumasa Koyanagi, Akihisa Terakita, Gebhard F.X. Schertler, Joachim Heberle, and Elena Lesca**

## Supplemental Material

### Figure S1

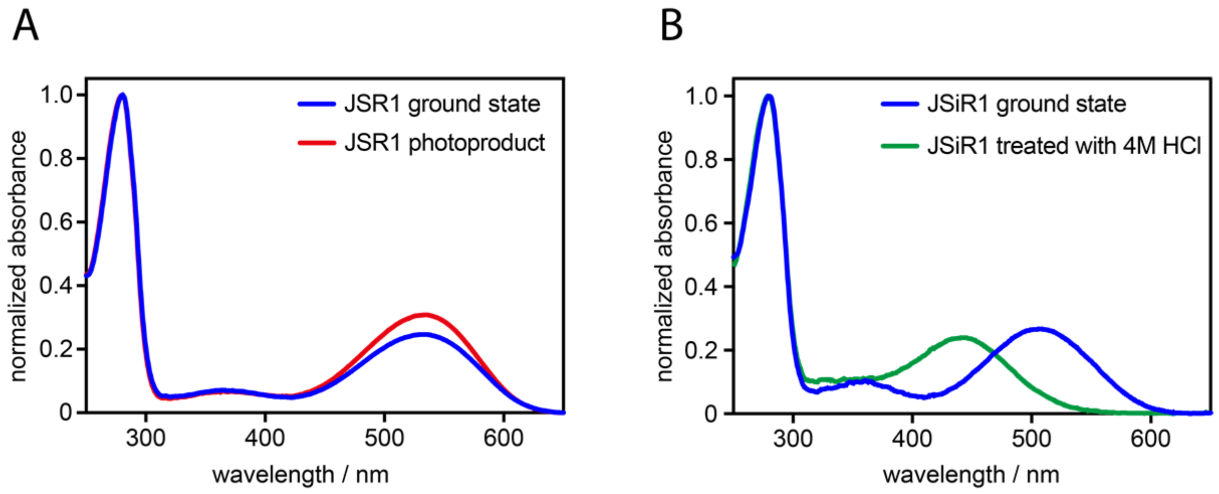


**Normalized UV/Vis spectra of JSR1**. **A)** JSR1 reconstituted with 11-*cis* retinal and of its photoproduct. They both have a maximum absorbance at 535 nm (16). **B)** UV/Vis absorption spectra of JSiR1 before and after acid denaturation. JSR1 reconstituted with 9-*cis* retinal was denaturated in the dark, resulting the typical ~440 nm maxima absorbance as indication of free Schiff base in solution (53).

### Figure S2

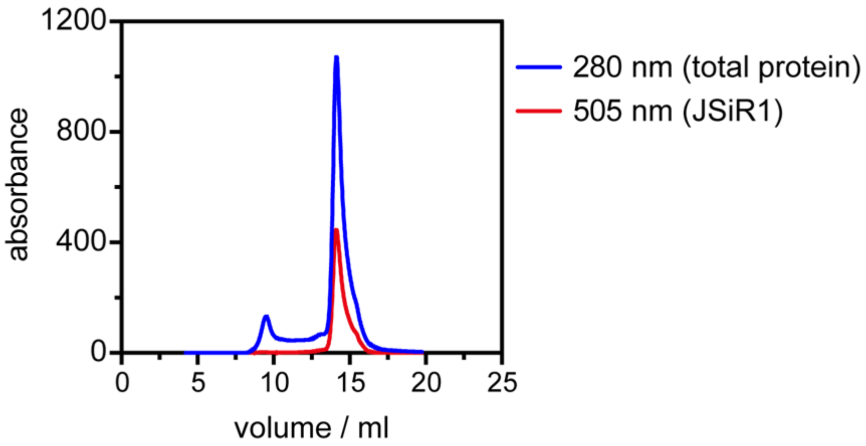


**Purification of recombinant expressed JSiR1.** Size-exclusion chromatography shows JSiR1 as a monodisperse sample. 280 nm absorbance (blue line) refers to the total protein amount, while 505 nm (red line) indicates the protonated Schiff base signal.

**Figure S3**

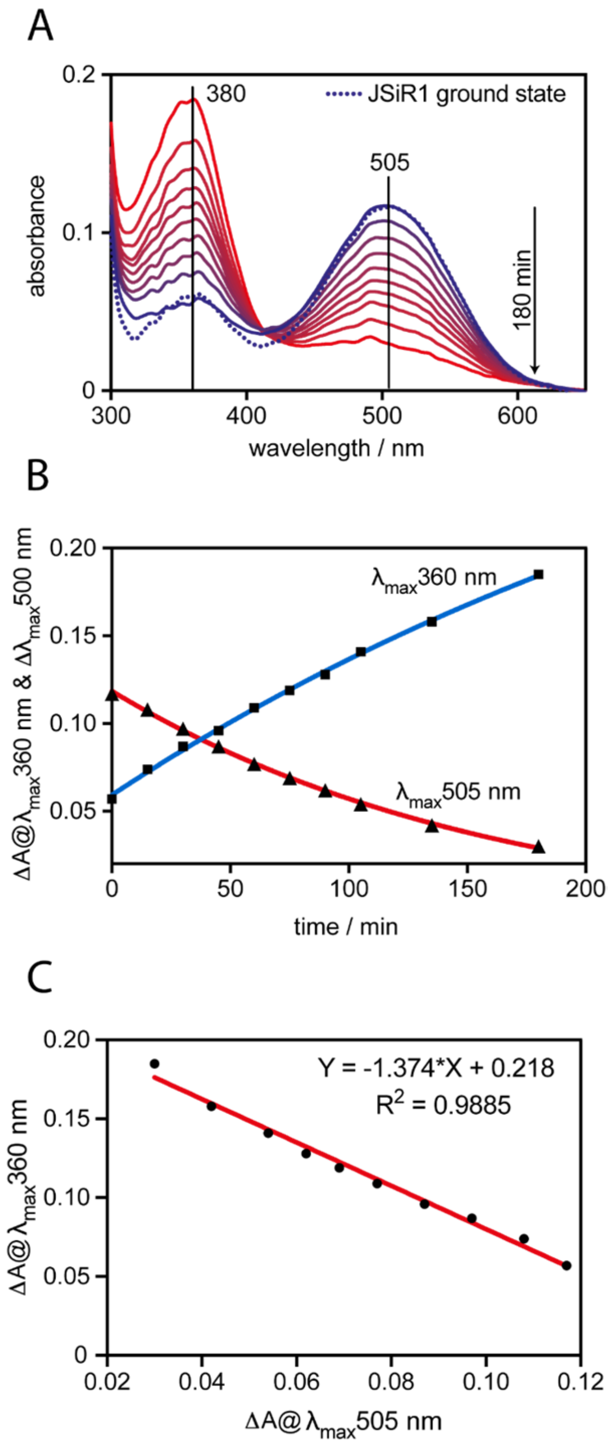


**Extinction coefficient of JSiR1 in the opsin buffer. A)** Hydroxylamine assay recorded over 3 hrs. The maxima absorbances at 360 nm (retinal) and 505 nm (JSiR1) are highlighted. **B)** The Δ360 and Δ505 are plotted against time (non-linear regression), showing a simultaneous increase/release of 9-*cis* retinal respectively **C)** Linear regression of Δ360 versus Δ505 shows the relation between increase/release of 9-*cis* retinal respectively. The slope corresponds to the Δ360/Δ505 = $\varepsilon_{retinal}/\varepsilon_{JSiR1}$, and has a value -1.37 ± 0.05.

**Figure S4**

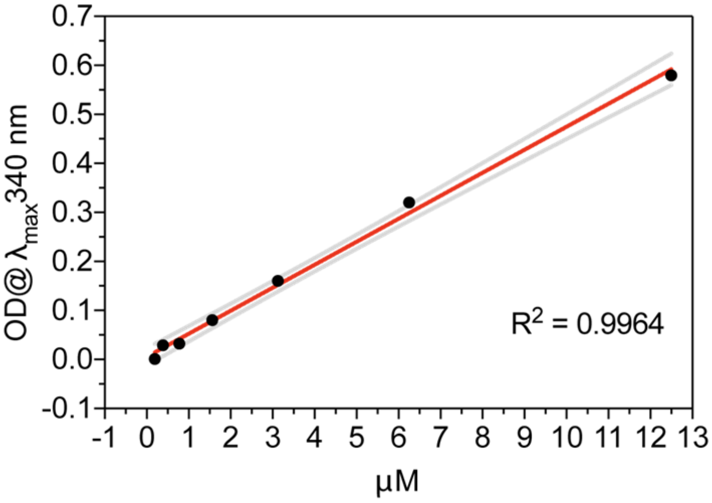


**Standard curve of 9-*cis* retinal oxime in the opsin buffer.** The $\varepsilon_{9\text{-}cis\ \text{retinal}}$ is 46930 $M^{-1}$ $cm^{-1}$.

**Figure S5**

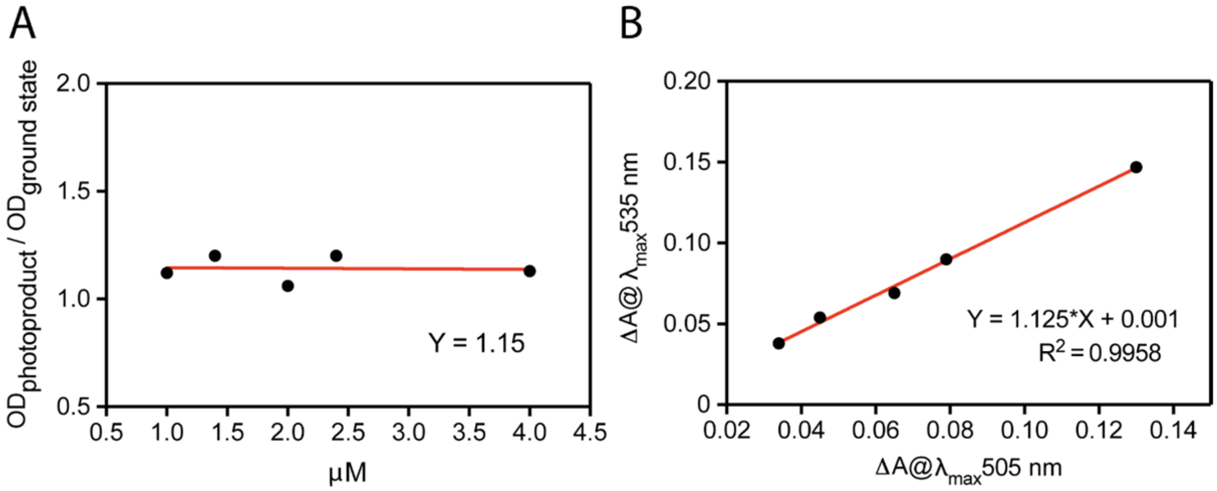


**Extinction coefficient of JSR1 photoproduct in the opsin buffer**. **A)** Interpolation of the ratio $OD_{photoproduct}/OD_{ground\ state}$ versus diverse concentration of JSiR1. The intercept corresponds to the $OD_{photoproduct}/OD = \varepsilon_{JSR1}/\varepsilon_{JSiR1}= 1.15$. **B)** Linear regression of OD max at 535 nm versus OD max at 505 nm. The slope corresponds to the $OD_{photoproduct}/OD_{ground\ state}$ and has a value 1.12 ± 0.04.

**Figure S6**

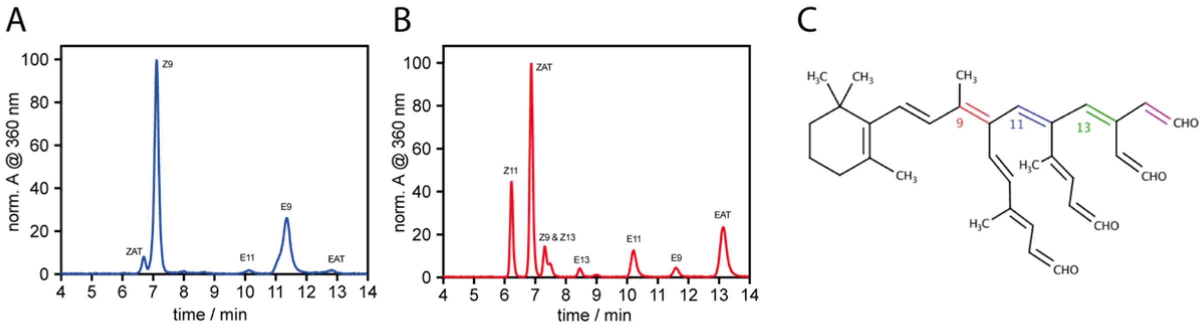


**Chromophore configurations of JSiR1 before and after irradiation**. HPLC chromatograms of retinal oximes (syn = Z, anti = E forms of retinal oximes) recorded at 360 nm and normalized **A)** JSiR1 ground state. **B)** JSR1 photoproduct. **C)** Chemical draw of retinal isomers.

The ground state of JSiR1 shows an interesting significative percentage of all-trans retinal (8%, Figure 2). Even if all the experiments were under controlled 640 nm light, we cannot exclude light-independent isomerization phenomena (e.g. thermal isomerisation) and, in fact, similar cases have been already reported. For instance, mouse melanopsin —30% similar to JSR1— shows thermal isomerization at 37˚C(1). Another example is the mekada TMT1A opsin, that also displays an heterogenous composition in the ground state (all *trans*, 9-*cis* and 13-*cis* retinal) (2).

1. Tsukamoto, H., Y. Kubo, D.L. Farrens, M. Koyanagi, A. Terakita, and Y. Furutani. 2015. Retinal attachment instability is diversified among mammalian melanopsins. J. Biol. Chem. 290: 27176–27187.
2. Sakai, K., T. Yamashita, Y. Imamoto, and Y. Shichida. 2015. Diversity of active states in TMT opsins. PLoS One. 10: 1–12.

**Figure S7**

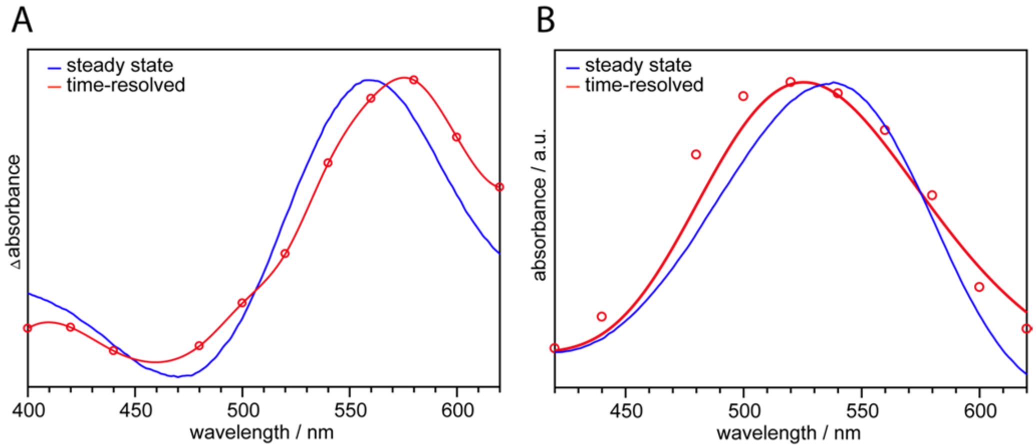


**Comparison of Flash Photolysis with Steady State data. A)** The shape of the final spectrum of the time-resolved dataset (red) resembles that of Meta-minus-Iso (blue). **B)** The absolute spectrum of a-Meta obtained by addition of the steady state spectrum of Iso to the time-resolved dataset has a slightly different shape compared to the Meta/Rho spectrum after illumination. This could indicate a different *syn*/*anti* composition due to the laser pulses. The difference in absorption maxima is less than 10 nm.

**Figure S8**

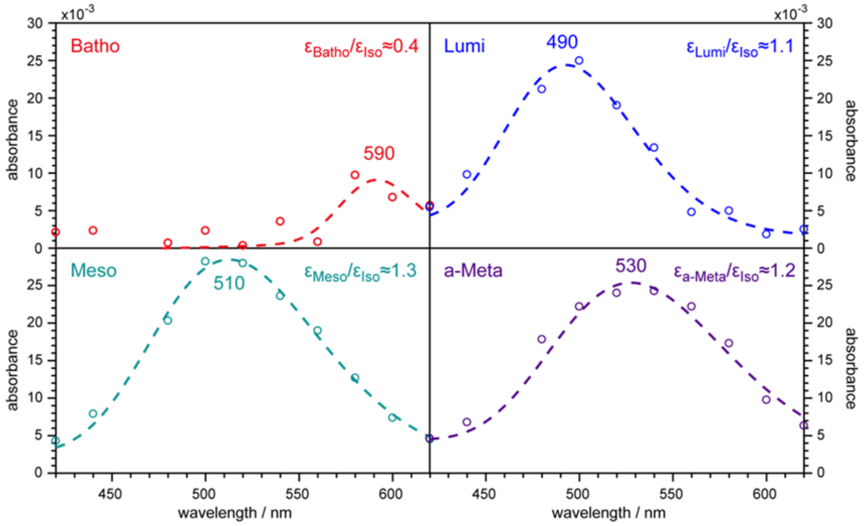


**Calculated absolute spectra of the photoproducts in the forward reaction.**
Absolute spectra of the intermediates in the forward photoreaction Iso to Meta. These were obtained by eliminating the negative contribution of Iso in the difference dataset.

It has to be noted that maxima in difference signals might not represent the real absorption maximum of the intermediate species due to spectral overlap of the initial state and the photoproduct. To determine the real absorption maxima of the respective intermediate states, the negative contribution of the initial Iso state in the dataset of the forward reaction must be subtracted. This is achieved by adding the UV/Vis spectrum of Iso in such a way that yields only positive absorption (58). Since the Meta state concludes the time-resolved dataset, the addition of the Iso spectrum was done by reproducing the spectral shape of the steady-state Meta spectrum obtained via UV/Vis spectroscopy (Figure S7B, a non-linear least squares regression was performed in MATLAB to approximate the steady-state spectrum of the illuminated sample (Figure S3B)). The resulting spectra of Batho, Lumi and Meso show absorption maxima at 590, 490 and 510 nm, respectively (Figure 4). Comparing the maximum absorption of the calculated intermediate spectra with the added Iso spectrum yields extinction coefficient ratios, which are typical for bistable pigments (59) except for the low extinction coefficient for Batho. The extinction coefficient ratio $\varepsilon_{Meta}/\varepsilon_{Iso}$ of 1.2 is in accordance the previously found value of 1.15 (see “Features of JSR isorhodopsin (JSiR1)”) confirming our approach.

**Figure S9**

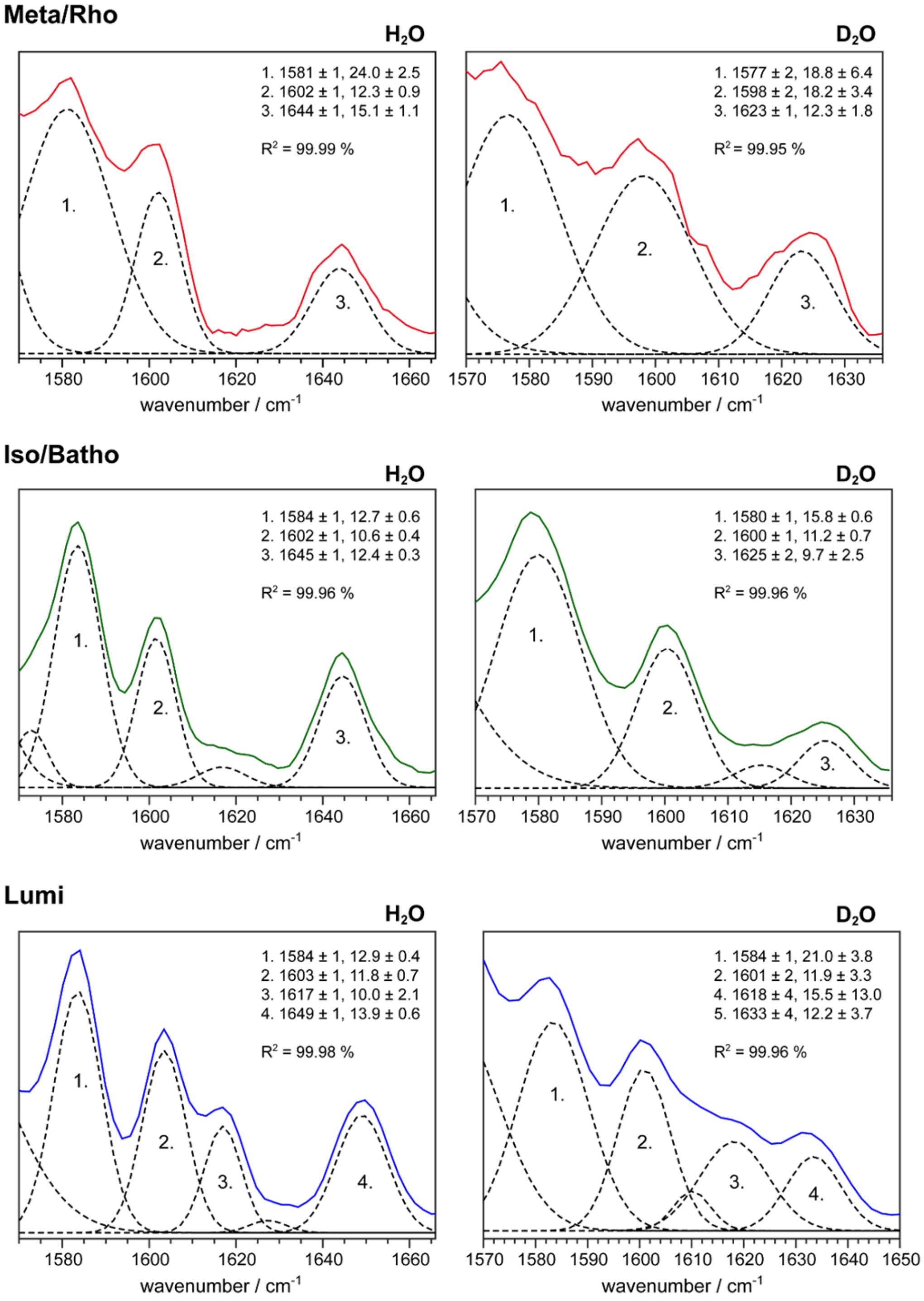


**Fits of the Schiff Base region.**
The dashed lines represent the fits with Gaussian functions on the raw data. R-squared was for all fits above 99.95 %. Fit parameters with corresponding error are given in the top right corner.

**Figure S10**

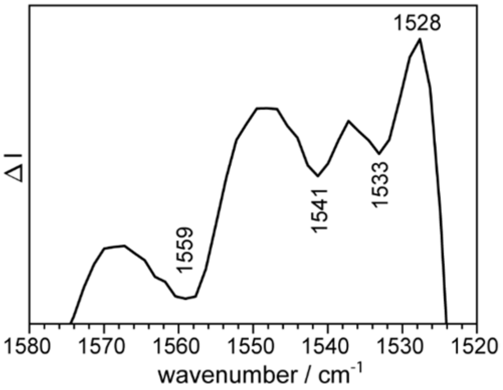


**Resonance Raman difference spectra of Meta/Rho minus Isorhodopsin in the C=C stretch region**
Spectra of Figure 7 were normalized to band at 1008 $cm^{-1}$ prior the subtraction. The resulting band pattern resembles the FTIR difference spectra of Figure 8.

**Figure S11**

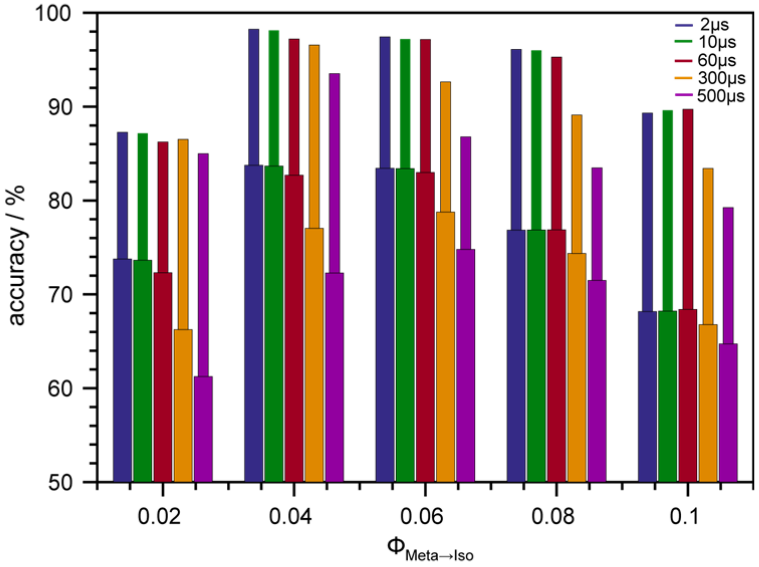


**Photostationary state simulation.**

The photolysis rate depends on the quantum yield(s) of a certain state in the photocycle of JSR1 as described in “Methods”. This value is unknown for Rho and Meta, as well as for other possible transitions from Meta-to-Iso. Hence, these values were varied from 0.5 to 0.9 and from 0.2 to 0.7, respectively. For each combination of these quantum yields the system of ODEs were solved for different time constants of the photoreverse reaction, i.e. from Meta to Rho. The plot shows the best scenario (thinner bar) and the overall performance (all combinations of quantum yields; wider bar) for a given Meta to Iso quantum efficiency and time constant for the photoreverse reaction. Best results are achieved for quantum efficiencies between 0.04 and 0.06 as well as for time constants below 60 µs. For larger time constants, the accuracy starts to drop significantly.

The table S1 grants a more detailed view of other parameters, which are grouped into the wider bar. An accuracy above 90% is considered to be in accordance with the HPLC data due to the experimental error in the determination of the retinal isomers and are highlighted in green for $\Phi_{Rho \to Iso}$ = 0.04 and in blue for 0.06. The corresponding value is the fraction of $\Phi_{Rho}/\phi_{Meta}$. For $\Phi_{Rho \to Iso}$ = 0.04 a fraction of at least 2 is needed in order to reproduce the HPLC results while for 0.06 the average fraction is 1.6. This value is in accordance to literature values for other bistable opsins. Overall, it can be stated that it is a prerequisite for Meta accumulation that the quantum yield of Meta is lower than that of Rho and that the photoreverse reaction (Meta -> Rho) has to be faster than the photoactivation (Rho -> Meta).

**Table S1**

| $\Phi_{Rho}/\phi_{Meta}$ | **0.2** | **0.3** | **0.4** | **0.5** | **0.6** | **0.7** |
|---|---|---|---|---|---|---|
| **0.5** | 2.5 | 1.7 | 1.3 | 1 | 0.8 | 0.7 |
| **0.6** | 3 | 2 | 1.5 | 1.2 | 1 | 0.9 |
| **0.7** | 3.5 | 2.3 | 1.7 | 1.4 | 1.2 | 1 |
| **0.8** | 4 | 2.7 | 2 | 1.6 | 1.3 | 1.1 |
| **0.9** | 4.5 | 3 | 2.3 | 1.8 | 1.5 | 1.3 |

Green: 0.04, blue: 0.06
